# The paradox of SMURF-less outcomes and its implication for diabetes

**DOI:** 10.1093/ehjqcco/qcag012

**Published:** 2026-01-28

**Authors:** Edina Cenko, Olivia Manfrini, Jinsung Yoon, Maria Bergami, Zorana Vasiljevic, Guiomar Mendieta, Marija Zdravkovic, Marija Vavlukis, Sasko Kedev, Davor Miličić, Filippo Ottani, Lina Badimon, Raffaele Bugiardini

**Affiliations:** Department of Medical and Surgical Sciences, University of Bologna, Via Massarenti 9, Bologna 40138, Italy; Department of Medical and Surgical Sciences, University of Bologna, Via Massarenti 9, Bologna 40138, Italy; IRCCS Azienda Ospedaliero-Universitaria di Bologna Sant’Orsola Hospital, Bologna, Italy; Google Cloud AI, Sunnyvale, CA, USA; Department of Medical and Surgical Sciences, University of Bologna, Via Massarenti 9, Bologna 40138, Italy; Medical Faculty, University of Belgrade, Belgrade, Serbia; Department of Cardiology, Hospital de la Santa Creu i Sant Pau, Barcelona, Spain and Centro Nacional de Investigaciones Cardiovasculares Carlos III (CNIC), Madrid, Spain; Faculty of Medicine, Clinical Hospital Center Bezanijska Kosa, University of Belgrade, Belgrade, Serbia; University Clinic for Cardiology Skopje, Republic of North Macedonia; Faculty of Medicine, Ss. Cyril and Methodius University in Skopje, Skopje, Republic of North Macedonia; University Clinic for Cardiology Skopje, Republic of North Macedonia; Faculty of Medicine, Ss. Cyril and Methodius University in Skopje, Skopje, Republic of North Macedonia; Department for Cardiovascular Diseases, University Hospital Center Zagreb, University of Zagreb, Zagreb, Croatia; Dipartimento di Cardiologia, Ospedale ‘Infermi’di Rimini, Rimini, Italy; University of Vic - UCC, School of Medicine and IRIS-CC Research Center, Vic; CiberCV, institute Carlos III; and Cardiovascular Research Foundation for Health and Innovation, Barcelona, Spain; Department of Medical and Surgical Sciences, University of Bologna, Via Massarenti 9, Bologna 40138, Italy

**Keywords:** Standardized modifiable risk factors, Coronary heart disease, Acute coronary syndromes, Mortality, Outcomes

## Abstract

**Aims:**

Individuals without standardized modifiable risk factors (SMuRF), which implicitly include those with diabetes, have been paradoxically reported to experience higher mortality following acute coronary syndromes (ACS). We aim to clarify the independent impact of diabetes on 30-day mortality after ACS and explore how grouping it with other SMuRF might obscure its true effect.

**Methods and results:**

We analyzed 70 953 first-time ACS patients using inverse probability weighting to adjust for potential confounding. Mortality within 30 days post-ACS was the primary outcome. Diabetic patients without other SMuRF showed a significantly higher 30-day mortality compared with those without any SMuRF, with relative risks (RRs) of 1.29 for women (95% CI, 1.06–1.57) and 1.40 for men (95% CI, 1.16–1.69). When diabetes was combined with other SMuRF, its impact on mortality was diluted. Diabetic patients who were also smokers had RRs of 1.39 in women (95% CI, 0.92–2.09) and 0.89 in men (95% CI, 0.68–1.17), those with hypercholesterolaemia had RRs of 0.91 in women (95% CI, 0.66–1.25) and 0.75 in men (95% CI, 0.53–1.06) and those with hypertension showed RRs of 1.14 in women (95% CI, 0.99–1.32) and 1.12 in men (95% CI, 0.96–1.31).

**Conclusion:**

Diabetes independently increases 30-day mortality risk in ACS. Aggregating it with other SMuRF masks this risk due to dilution bias, highlighting the need for individualized risk factor assessment strategies.

## Graphical Abstract

**Graphical Abstract:**
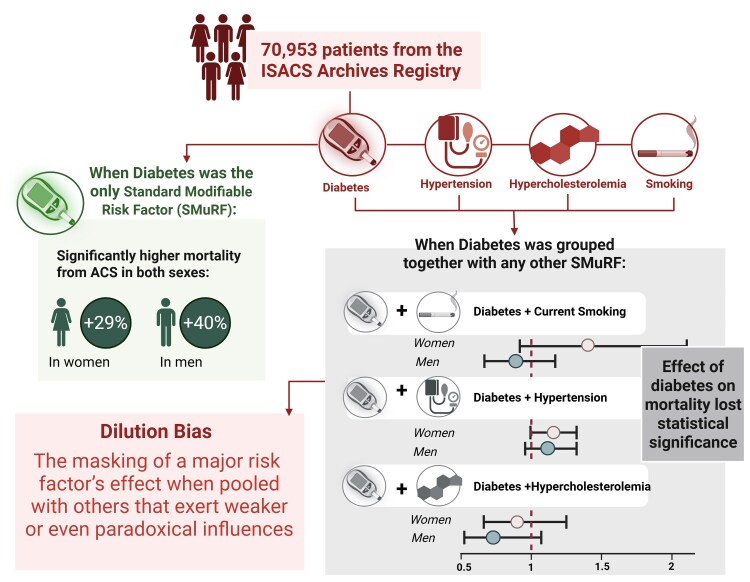
Breakdown of Dilution Bias and the main findings for each SMuRF subgroup. ACS, acute coronary syndrome; SMuRF, standard modifiable cardiovascular risk factor. Figures made on Biorender.com

Key Learning Points What is already known:

- This study challenges claims of higher mortality from acute coronary syndromes (ACS) in patients without SMuRF.

What this study adds:

- Diabetes significantly increases 30-day mortality post-ACS.
- Dilution bias occurs when diabetes is combined with other SMuRFs
- Pooling SMuRFs together risks unaccounted confounding and effect modification
- Current ACS risk prediction tools may need revision to include the impact of diabetes.

## Introduction

Acute coronary syndromes (ACS) remain a leading cause of mortality worldwide.^1^ While standardized modifiable risk factors (SMuRF), current smoking, diabetes, hypercholesterolaemia, and hypertension are well-established contributors to the development of ACS, their impact on ACS outcomes, especially short-term mortality, remains complex.

Recent studies have paradoxically reported higher short-term mortality in ACS patients who lack SMuRF.^2-7^ This ‘SMuRF-less paradox’ challenges conventional views regarding diabetes, a metabolic disorder that markedly worsens cardiovascular outcomes.^8-10^ These investigations have grouped patients with one or more SMuRFs into a single category, assuming that all risk factors exert similar effects. Such aggregation overlooks the unique clinical significance of diabetes, whose mechanisms—chronic hyperglycaemia, endothelial dysfunction, and microvascular injury- differ fundamentally from those of lifestyle-related risks such as smoking or dyslipidaemia. This practice can obscure diabetes’ true impact on post-ACS mortality, introducing dilution bias, whereby strong effects of one factor are masked by weaker or opposing effects of others.

On this background, we aimed to determine the independent effect of diabetes on 30-day mortality after ACS, both as a solitary risk factor and in combination with other SMuRFs. By disentangling these relationships, our study seeks to reconcile conflicting findings in the literature and reaffirm the importance of recognizing diabetes as a distinct and consistently high-risk clinical profile in the acute coronary setting.

## Methods

### Study subjects

The study population consisted of 70 953 Caucasian patients enrolled in the International Survey of Acute Coronary Syndromes (ISACS) Archives (NCT04008173) registry network for a first manifestation of ACS from October 2005 to January 2021 (see Supplementary material online, *Figure S1*). Patients with prior coronary heart disease (CHD) or heart failure of unknown origin were excluded. The design of the ISACS Archives has been previously described.^11-13^ Details of the study sampling and recruitment are summarized in the Supplementary data. The local research ethics committee from each hospital approved the study. Because patient information was collected anonymously, institutional review boards waived the need for individual-informed consent. This study complies with the Declaration of Helsinki. All data were transferred to the Department of Electrical and Computer Engineering, University of California, Los Angeles, where final statistical analyses were done.

### Study design

Clinical data were collected from hospital records by trained abstractors following a standardized protocol, utilizing physician notes, laboratory reports, and patient medical histories. Based on previous studies, we classified current smoking, hypertension, diabetes, and hypercholesterolaemia as SMuRFs for CHD. In line with prior research, we grouped patients with one or more of these factors into a single category labelled ‘patients with one or more SMuRF.’ However, unlike previous investigations, we also analysed the individual strength of association between each risk factor and the outcomes of interest, without relying on a cumulative definition.

### Outcome measures

The primary outcome measure was all-cause mortality within 30 days of hospital admission. The 30-day window was selected to enrich the data over that acquired during the index hospitalization while mitigating survivor bias. Coronary artery bypass grafting (CABG) was always performed as an urgent surgical intervention following percutaneous coronary intervention (PCI). Therefore, outcomes of CABG procedures were included in the subgroup of patients undergoing PCI revascularization.

### Concomitant care and definitions

We noted the type of medications given on hospital admission, during hospitalization, and discharge. All patients with a glomerular filtration rate <60 mL/min/1.73 m^2^ for 3 months were defined as having chronic kidney disease.^14^ Risk factors for CHD were identified during hospitalization, as documented in the medical record, and were based on patient self-report or previous medical records (see Supplementary data). Due to its critical role in the management of ST-segment elevation myocardial infarction (STEMI), we categorized the time to hospital presentation as a dichotomous variable: delayed (≥120 min) vs. early (<120 min), following the American College of Cardiology (ACC)/American Heart Association (AHA) practice guidelines.^15^ This categorization was not applied to patients with non-ST-segment elevation acute coronary syndromes (NSTE-ACS), where the timing of presentation has less immediate clinical implications for guiding acute management strategies.

### Statistical analysis

Patients were categorized according to their type of SMuRF. We specifically examined the effects of diabetes both in isolation and in combination with other SMuRFs. Subgroups were stratified by sex. Baseline characteristics were reported as number (percentages) for categorical variables and mean ± standard deviation (SD) for continuous variables. We had complete data on mortality, sex, age, and index event. Missing data, which ranged from 9.8% to 18.1%, were addressed using multiple imputation by chained equations (MICE).^16^ To mitigate potential confounding and selection bias, we employed inverse probability weighting (IPW) based on propensity scores to balance patient characteristics across groups^17^ Standardized differences (SD) after weighting were computed after weighting to verify covariate balance; groups were considered adequately balanced when SDs were <10%^18^ The baseline covariates included in the IPW models comprised demographic variables, cardiovascular risk factors, prior cardiovascular disease, and clinical features at hospital presentation (*Table 1*) Because extreme weights can produce unstable or biased estimates, we conducted sensitivity analyses to test robustness. Specifically, we compared the IPW results with those obtained using XGBoost, a decision-tree–based ensemble machine-learning algorithm, which provides a flexible approach to model confounding structures. The conclusions from these analyses were consistent with the main findings, confirming the robustness of our results. To evaluate the robustness of our findings, we conducted sensitivity analyses across major therapeutic subgroups, stratifying outcomes according to reperfusion or revascularization modality (PCI, fibrinolysis, or CABG), timing of presentation (≤2 h vs. >2 h), and use of key pharmacologic treatments (aspirin/P2Y_12_ inhibitors, heparin, and glycoprotein IIb/IIIa inhibitors). Results of these analyses, summarized in the Supplementary data, were consistent with the primary findings, confirming that the observed associations between diabetes as a solitary risk factor and 30-day mortality were stable across treatment and timing strata. Risk ratios (RRs) with their corresponding 95% confidence intervals (CIs) were calculated in the weighted population to estimate associations between SMuRF and outcomes. To minimize concern about the comparison of outcomes in subgroups, estimates were compared by a test of interaction on the log scale.^19^ A *P* value <0.05 was taken to indicate that the difference between the outcomes in subgroups was unlikely to have occurred simply by chance. A detailed description of the statistical methods and adjustments is provided in the Supplementary data.

**Table 1:** Inverse probability of weighting: outcomes stratified by sex and SMURFS status

Characteristics	Women			Men		
	SMuRFs (*N* = 21 451)	SMuRF-less (*N* = 4039)	Standardized difference	SMuRFs (*N* = 38 442)	SMuRF-less (*N* = 7021)	Standardized difference
Age (years)	66.4 ± 11.3	66.4 ± 12.3	−0.0003	60.9 ± 11.7	60.5 ± 12.6	0.0335
**Cardiovascular risk factors**						
Family history of CAD	30.0	30.0	0.0015	29.4	29.5	−0.0034
Former smokers	0.8	0.8	0.0007	2.3	2.6	−0.0168
BMI ≥30 kg/m^2^	18.7	18.9	−0.0040	20.3	20.7	−0.0081
**Clinical history of CVD**						
Peripheral artery disease	2.3	2.0	0.0223	2.4	2.2	0.0097
Prior stroke	4.2	4.1	0.0028	3.7	3.9	−0.0106
**Clinical presentation on admission**						
ST-segment shifts in anterior leads (at ECG)	20.2	20.4	−0.0051	21.8	22.0	−0.0048
SBP at admission (mmHg)	137.6 ± 29.0	137.9 ± 30.9	−0.0137	138.7 ± 28.1	138.7 ± 29.2	−0.0183
HR at admission (bpm)	82.5 ± 20.3	82.7 ± 21.0	−0.0094	81.2 ± 19.7	81.2 ± 19.6	0.0002

Outcomes			*P* value			*P* value
30-day mortality	11.0	14.8	<0.0001	6.4	9.6	<0.0001
Risk Ratio (95% CI)	0.72 (0.65–0.79)		<0.0001	0.64 (0.59–0.70)		<0.0001

BMI, body mass index; CAD, coronary artery disease; CVD, cardiovascular disorders; ECG, electrocardiogram; HR, heart rate; SBP, systolic blood pressure; SMuRF, standard modifiable cardiovascular risk factor.

Data are presented as percentages (%) or mean ± standard deviation, unless otherwise specified.

## Results

### Baseline characteristics of patients

Among ACS patients, ≥1 SMuRFs were present in 84.2% of women and 84.6% of men (see Supplementary material online, *Figure S2*). Women had a higher prevalence of all SMuRFs except smoking (see Supplementary material online, *Table S1*). SMuRF-less patients were less likely to undergo invasive procedures and received fewer guideline-directed therapies.

### Overall outcomes

Compared with SMuRF-less patients, those with ≥1 SMuRF had lower 30-day mortality (women: 11.0% vs. 14.8%, RR 0.72 [95% CI 0.65–0.79]; men: 6.4% vs. 9.6%, RR 0.64 [95% CI 0.59–0.70]; *P*-interaction = 0.04) (*Table 1* and Supplementary material online, *Table S2*). Overall, the absence of SMuRFs was associated with an approximately 30% lower likelihood of 30-day mortality (RR, 0.68; CI, 0.64–0.73) (see Supplementary material online, *Table S3*). This association persisted after adjustment for treatments received (see Supplementary material online, *Tables S4*  to  *S9*).

### Diabetes as an isolated risk factor

**Figure 1:**
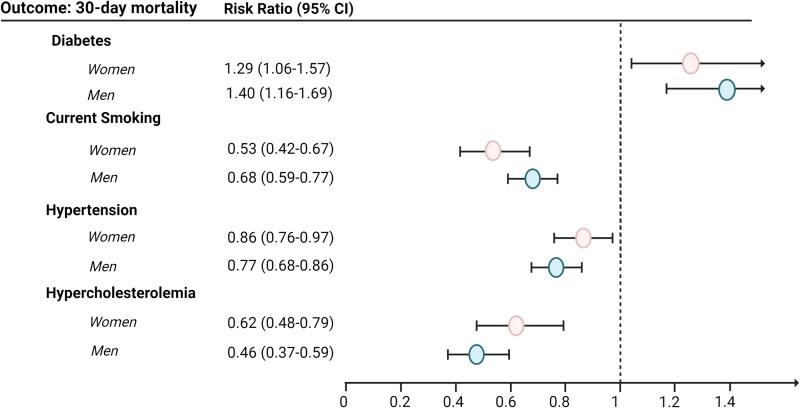
Inverse probability of treatment weighting models: effects on outcomes of each of the four SMuRF (current smoking status, hypertension, or hypercholesterolaemia) compared with the absence of SMuRF, stratified by sex. CHD, coronary heart disease; SMuRF, standard modifiable cardiovascular risk factor. Figures made on Biorender.com.

Patients with diabetes as their only SMuRF (*Figure 1*; *Table 2* and Supplementary material online, *Table S10*) had markedly higher 30-day mortality than those with no SMuRFs (women: RR 1.29 [95% CI 1.06–1.57]; men: 1.40 [95% CI 1.16–1.69]). The association remained robust across treatment subgroups (see Supplementary material online, *Tables S11*  to  *S16*).

**Table 2:** Inverse probability weighting: outcomes stratified by sex and SMURFS status. Comparison between patients with diabetes as a solitary risk factor and those without any SMuRFs

	Women			Men		
Characteristics	Diabetes (*N* = 731)	SMuRF-less (*N* = 4039)	Standardized difference	Diabetes (*N* = 960)	SMuRF-less (*N* = 7021)	Standardized difference
Mean ± SD age, years	67.9 ± 11.3	67.5 ± 11.9	0.0353	63.5 ± 10.7	63.1 ± 12.2	0.0330
**Cardiovascular risk factors**						
Family history of CAD, %	16.2	14.9	0.0345	13.9	14.5	−0.0189
Former smokers, %	0.5	0.6	−0.0029	2.3	2.3	0.0029
BMI ≥30 kg/m^2^, %	11.2	11.6	−0.0116	14.1	14.2	−0.0027
**Clinical history of CVD**						
PAD, %	1.5	1.4	0.0035	1.5	1.5	−0.0016
Prior stroke, %	3.4	3.3	0.0015	3.0	3.0	−0.0023
**Clinical presentation on admission**						
ST-segment shifts in anterior leads (at ECG), %	19.2	18.4	0.0222	20.2	20.1	0.0037
Mean ± SD SBP at admission, mmHg	129.0 ± 30.5	129.5 ± 30.1	−0.0144	132.1 ± 28.8	131.9 ± 29.3	0.0076
Mean ± SD HR at admission, bpm	82.2 ± 19.5	81.9 ± 20.8	0.0184	79.9 ± 20.9	80.3 ± 19.6	−0.0214

Outcomes			*P* value			*P* value
30-day mortality, %	20.7	16.8			15.9	11.9
Risk Ratio (95% CI)	1.29 (1.06–1.57)	0.0124		1.40 (1.16–1.69)	0.0005	

BMI, body mass index; CAD, coronary artery disease; CHD, coronary heart disease; CVD, cardiovascular disorders; ECG, electrocardiogram; HR, heart rate; PAD, peripheral artery disease; SBP, systolic blood pressure; SMuRF, standard modifiable cardiovascular risk factor.

Data are presented as percentages (%) or mean ± standard deviation, unless otherwise specified.

### Other individual SMuRFs

In contrast, smoking, hypercholesterolaemia, and hypertension, each in isolation, were associated with lower short-term mortality (*Figure 1*; Supplementary material online, *Tables S17*  to  *S19*).

### Diabetes and dilution bias

**Figure 2:**
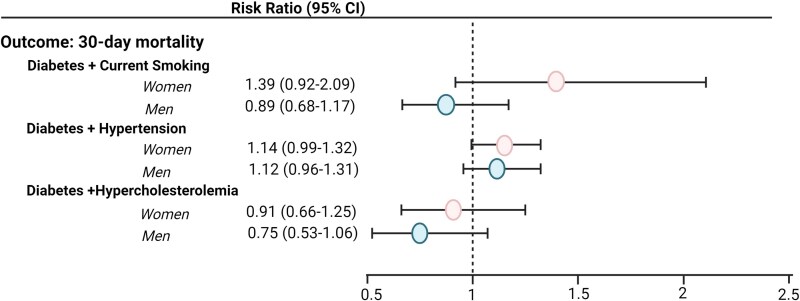
Example of dilution bias using inverse probability of treatment weighting models: effects on outcomes of diabetes combined separately with each of the three remaining SMuRF (current smoking status, hypertension, or hypercholesterolaemia) compared with the absence of SMuRF, stratified by sex. CHD, coronary heart disease; SMuRF, standard modifiable cardiovascular risk factor. Figures made on Biorender.com.

When diabetes coexisted with other SMuRFs, its effect on mortality was attenuated and became nonsignificant (*Figure 2* and Supplementary material online, *Tables S20*  to  *S22*). This attenuation, evident across combinations with smoking, hypercholesterolaemia, or hypertension, demonstrates dilution bias, wherein protective or neutral factors obscure the detrimental impact of diabetes (*Central Illustration*; *Figure 3*).

### Interaction test

To quantify whether the mortality risk associated with diabetes was attenuated in the presence of additional SMuRFs, we compared relative risks using the Altman test of interaction. (see Supplementary material online, *Table S23*). In men, the diabetes-related excess risk was significantly weakened when diabetes coexisted with either smoking (ratio of RRs = 1.58; 95% CI 1.13–2.19; *P* = 0.004), hypercholesterolaemia (1.87; 95% CI 1.26–2.77; *P* < 0.001), or hypertension (1.25; 95% CI 0.99–1.60; *P* = 0.04). In women, no statistically significant interaction was detected for most combinations, although a modest attenuation trend emerged for diabetes with hypercholesterolaemia (ratio 1.42 [0.97–2.06]; *P* = 0.04). These findings indicate that, particularly among men, the prognostic impact of diabetes becomes statistically diluted when additional SMuRFs are present, consistent with the concept of dilution bias.

## Discussion

The current study clarifies the relationship between diabetes and 30-day mortality following ACS, providing further evidence that diabetes independently increases the risk of death.^8-10^ Our findings challenge recent reports suggesting that patients without SMuRF have worse outcomes^4,6^ than those with SMuRF and show that such observations likely result from ‘dilution bias’, the masking of a major risk factor’s effect when pooled with others that exert weaker or even paradoxical influences.

Misinterpreting this phenomenon may lead both patients and clinicians to underestimate the danger of diabetes in the acute setting. Diabetic patients might wrongly assume their condition is less threatening, and clinicians might discount its prognostic weight. This issue therefore requires clarification.

Formal interaction testing supports this interpretation. The attenuation of diabetes-related mortality risk in the presence of additional SMuRFs, particularly smoking and hypercholesterolaemia in men, quantitatively demonstrates the dilution bias effect. When diabetes coexists with other factors that may show paradoxically neutral or protective short-term associations during the acute phase of ACS, its adverse prognostic signal becomes statistically weakened rather than clinically absent. Diabetes thus remains a dominant determinant of early mortality, but its contribution is obscured by heterogeneity in coexisting risk profiles. Recognizing this statistical dilution is crucial to avoid underestimating the clinical severity of diabetes in ACS and to ensure appropriately intensive management.

Biologically, diabetes likely worsens short-term outcomes through distinct pathophysiological mechanisms. Chronic hyperglycaemia induces endothelial dysfunction, impairs coronary microvascular flow, and promotes pro-inflammatory and pro-thrombotic states that amplify myocardial injury during acute ischaemia. It also blunts the benefits of ischaemic preconditioning and increases susceptibility to heart failure through diabetic cardiomyopathy.

In contrast, the seemingly ‘protective’ associations observed for hypertension, hypercholesterolaemia, and current smoking are most probably paradoxical rather than causal. These patients often reach medical attention earlier, are more readily recognized as cardiac cases, and receive more prompt reperfusion and guideline-directed therapies, including statins, β-blockers, and ACE inhibitors. Higher admission blood pressure may transiently preserve coronary perfusion, and prior statin exposure or chronic smoking may elicit preconditioning effects. These short-term advantages do not counteract the long-term harm of these factors but rather reflect differences in haemodynamic response, clinical suspicion, and treatment intensity at the time of presentation.

Our granular analysis, isolating the contributions of each individual risk factor, revealed a strong association between diabetes as a solitary SMuRF and increased 30-day mortality in ACS for both men (RR: 1.40; 95% CI: 1.16–1.69) and women (RR: 1.29; 95% CI: 1.06–1.57). These results parallel prior evidence from GUSTO-1 (11.3% vs. 5.9% mortality in diabetic vs. non-diabetic patients) and from pooled TIMI trials showing nearly a two-fold higher risk of death in diabetic patients post-ACS^8^,^20^ These results confirm the adverse prognostic role of diabetes, yet they also raise the question of why SMuRF-less patients appear to fare worse in other reports. Exploring potential contributors to this paradox is therefore essential.

Differences in early management have been suggested to contribute^4^. SMuRF-less patients might initially be misclassified as having non-cardiac conditions, leading to delayed diagnosis and reperfusion. However, our analyses adjusted for treatment-related variables, including time from symptom onset to reperfusion, type of reperfusion or revascularization (PCI, fibrinolysis, or CABG), and use of evidence-based pharmacotherapies, yet the mortality gap persisted. Treatment bias alone therefore cannot explain our findings, yet differences in recognition, triage, and care quality may still play a contributory role.

Beyond potential differences in treatment, ‘*dilution bias’* offers a plausible, though not exclusive, explanation of our findings. Pooling diabetic patients under the broad category of ‘one or more SMuRFs.’ can mask the effect of diabetes when combined with other SMuRFs, such as hypercholesterolaemia, current smoking, and hypertension, factors that may behave paradoxically in the acute phase of ACS, as our study and others have suggested.

Our analysis showed that hypercholesterolaemia as a solitary risk factor was associated with better outcomes, with RRs of 0.46 (95% CI: 0.37–0.59) in men and 0.62 (95% CI: 0.48–0.79) in women, in line with CRUSADE data showing lower mortality even after accounting for prior statin use (OR: 0.74; 95% CI: 0.68–0.80).^21^ A similar ‘smoker’s paradox’ has been reported in large registries,^22^ and higher blood pressure at presentation has been linked to reduce in-hospital mortality.^23,24^

These paradoxical associations underscore the clinical heterogeneity embedded within composite SMuRF categories. Against this background, diabetes stands apart. Its detrimental influence in the acute phase is consistent, independent, and biologically plausible, reflecting its established link with impaired myocardial reserve and diabetic cardiomyopathy. When aggregated with other SMuRFs, this consistent signal becomes attenuated; when examined in isolation, diabetes clearly identifies a population at high clinical risk requiring intensified management and vigilant follow-up.

### Strengths and limitations of the current study

Strengths of the current study include several areas, which contribute to the robustness and reliability of our findings. First, the study benefits from a large sample size of over 70 000 ACS patients, spanning a 16-year period. This extensive dataset allows for a more comprehensive analysis and increases the statistical power, making the results more generalizable to the broader population of ACS patients. Another key strength is the use of inverse probability weighting models to adjust for potential confounders and minimize bias. This statistical approach helps to address confounding by indication and reducing the impact of collider bias. Our study is also distinguished by its granular analysis of SMuRF, which includes an examination of each risk factor both individually and in combination. This approach enables a clearer understanding of how specific risk factors contribute to short-term mortality outcomes.

The study also has several limitations. As an observational analysis, it is inherently susceptible to potential bias and confounding. Inverse probability weighting was used to minimize these effects by balancing observed covariates across groups. However, residual confounding cannot be completely excluded, as detailed information on diabetes duration, glycaemic control, and antidiabetic treatment type was not available, which limits our ability to account for the heterogeneity of diabetes severity and management. Information bias may have occurred because some risk factors were obtained from general practitioners’ records or patient self-report, potentially leading to misclassification. The true prevalence of traditional risk factors is therefore likely higher than reported, since approximately 30% of patients with hypertension, hypercholesterolaemia, or diabetes remain undiagnosed.^25,26^ Selection bias may have occurred, as patients with prior coronary heart disease or heart failure were excluded; nevertheless, this ensured the inclusion of first-time ACS presentations. Reverse causation may likewise have influenced our findings if patients with very severe or rapidly fatal presentations died before their risk factors could be fully identified or documented, resulting in misclassification as SMuRF-less and introducing a potential source of bias that should be acknowledged when interpreting these estimates. Some caution is also warranted when interpreting the sex-specific interaction analyses, as these exploratory assessments may have limited statistical power. In addition, our findings should be extrapolated with care to other ACS populations or to longer follow-up periods.^27^ Finally, the study cohort consisted entirely of European White patients, which limits the generalizability of these findings to other ethnic groups. Application of the observed risk estimates to other race/ethnic groups may yield uncertain results. In particular, South Asian and African ancestry groups experience higher rates of diabetes- and hypertension-related cardiovascular complications, while risk-factor awareness and treatment intensity vary substantially across regions.

## Conclusions

Diabetes emerges as a distinct and consistently high-risk clinical profile in acute coronary syndromes. Its detrimental effect on 30-day mortality is consistent and independent, yet becomes statistically diluted when aggregated with other SMuRFs. This misclassification may conceal the true vulnerability of diabetic patients and lead to under-recognition of their risk.

Current management and prediction models should therefore treat diabetes not as one of several modifiable factors but as a *primary determinant* of early mortality after ACS. Tailored therapeutic strategies and intensive secondary prevention are warranted. Our findings call for an update of existing ACS risk-stratification tools, where diabetes should be explicitly re-evaluated as a defining high-risk condition.

## Supplementary Material

qcag012_Supplementary_Data

## Data Availability

To guarantee the confidentiality of personal and health information, only the authors have had access to the data during the study. The source codes for this manuscript are uploaded on GitHub: https://github.com/jsyoon0823/Treatment_Phenotype
