# Supplementary material for: The paradox of SMURF-less outcomes and its implication for diabetes
Source: Eur Heart J Qual Care Clin Outcomes. 2026 Jan 28;12(4):579–86. doi: 10.1093/ehjqcco/qcag012 (PMC13288731; doi:10.1093/ehjqcco/qcag012)
Supplement: qcag012_Supplementary_Data [file qcag012_supplementary_data.docx]

**Supplementary Data**

**The Paradox of SMURF-less Outcomes and its Implication for Diabetes.**

**Table of contents**

[**SUPPLEMENTAL METHODS** 4](#_Toc213598762)

[**The International Survey of Acute Coronary Syndromes (ISACS) Archives**. 4](#_Toc213598763)

[**Information on Registries of the ISACS- Archives** 4](#_Toc213598764)

[**Definition of risk factors for CHD** 5](#_Toc213598765)

[**Multiple Imputation using Chained Equation (MICE) algorithm.** 6](#_Toc213598766)

[**Inverse Propensity Score Weighting Analysis** 6](#_Toc213598770)

[**Computation of Relative Risk and its Confidence Interval** 7](#_Toc213598771)

[**Comparison of means and prevalences in the weighted sample** 9](#_Toc213598772)

[**Interaction test** 9](#_Toc213598773)

[**Interaction and trend analyses.** 10](#_Toc213598774)

[**SUPPLEMENTAL RESULTS** 11](#_Toc213598775)

[**Figure S1.** Study flow chart 11](#_Toc213598776)

[**Figure S2.** Prevalence of SMuRF among men and women hospitalized for acute coronary syndromes 12](#_Toc213598777)

[**Table S1. Baseline characteristics** 14](#_Toc213598778)

[**Table S2.** Interaction test: calculations for comparing two estimated risk ratios (women vs men) for 30-day mortality by inverse probability weighting: SMuRFs versus SMuRFs less patients 17](#_Toc213598779)

[**Table S3.** Inverse probability weighting: clinical factors and outcomes stratified by SMuRFs status 18](#_Toc213598780)

[**Table S4.** Inverse probability weighting: clinical factors and outcomes stratified by SMuRFs status in patients undergoing reperfusion therapy for **STEMI: PCI, fibrinolysis, or CABG**. 20](#_Toc213598781)

[**Table S5.** Inverse probability of weighting: outcomes stratified by SMuRFs status and time from symptom onset to hospital admission in patients undergoing reperfusion therapy for **STEMI (**PCI, fibrinolysis or CABG) 21](#_Toc213598782)

[**Table S6.** Inverse probability weighting: outcomes stratified by SMuRFs 23](#_Toc213598783)

[status in patients undergoing **revascularization therapy (PCI or CABG) in NSTE-ACS** 23](#_Toc213598784)

[**Table S7.** Inverse probability weighting: outcomes stratified by SMuRFs status in **aspirin and/or P2Y_12_ inhibitors users** 24](#_Toc213598785)

[**Table S8.** Inverse probability weighting: outcomes stratified by SMuRFs status in **heparin users** 25](#_Toc213598786)

[**Table S9**. Inverse probability weighting: outcomes stratified by SMuRFs status in Glycoprotein IIb/IIIa inhibitors users 27](#_Toc213598787)

[**Table S10.** **Interaction test**: calculations for comparing two estimated risk ratios (women vs men) for 30-day mortality by inverse probability weighting: diabetes as their sole SMuRF versus SMuRF-less. 28](#_Toc213598788)

[**Table S11.** Inverse probability weighting: outcomes stratified by SMURFS status. Comparison between patients with diabetes as a solitary risk factor and those without any SMuRFs in the subgroup undergoing reperfusion therapy for **STEMI (**PCI, fibrinolysis, or CABG). 29](#_Toc213598789)

[**Table S12**. Inverse probability weighting: Patients undergoing reperfusion therapy for **STEMI** (PCI, fibrinolysis, or CABG) at different times from symptom onset to hospital admission (≤2 hours vs **>** hours). Comparison between patients with diabetes as a solitary risk factor and those without any SMuRFs 30](#_Toc213598790)

[**Table S13.** Inverse probability weighting: Patients undergoing reperfusion therapy for **NSTE-ACS** (PCI, fibrinolysis, or CABG). Outcomes stratified by SMURFS status. Comparison between patients with diabetes as a solitary risk factor and those without any SMuRFs 32](#_Toc213598791)

[**Table S14.** Inverse probability weighting: and outcomes sorted by SMURFS status. Comparison between patients with diabetes as a solitary risk factor and those without any SMuRFs in the subgroup undergoing heparins’use. 33](#_Toc213598792)

[**Table S15.** Inverse probability weighting: Outcomes sorted by SMURFS status. Comparison between patients with diabetes as a solitary risk factor and those without any SMuRFs in the subgroup undergoing a**spirin and/or P2Y_12_ inhibitors use** 34](#_Toc213598793)

[**Table S16 .** Inverse probability weighting: Outcomes stratified by SMURFS status. Comparison between patients with diabetes as a solitary risk factor and those without any SMuRFs in the subgroup administered Glycoprotein IIb/IIIa inhibitors. 36](#_Toc213598794)

[**Table S17.**Inverse probability weighting: outcomes stratified by sex and SMURFS status. Comparison between patients with current smoking as a solitary risk factor and those without any SMuRFs. 38](#_Toc213598795)

[**Table S18.** Inverse probability weighting: outcomes stratified by sex and SMURFS status. Comparison between patients with hypertension as a solitary risk factor and those without any SMuRFs. 40](#_Toc213598796)

[**Table S19.** Inverse probability weighting: Outcomes stratified by sex and SMURFS status. Comparison between patients with hypercholesterolemia as a solitary risk factor and those without any SMuRFs. 42](#_Toc213598797)

[**Table S20**. Inverse probability weighting: outcomes stratified by sex and SMURFS status. Comparison between patients with both diabetes and current smoking as concurrent risk factors and those without any SMuRFs 44](#_Toc213598798)

[**Table S21.** Inverse probability weighting: outcomes stratified by sex and SMURFS status. Comparison between patients with both diabetes and hypercholesterolemia as concurrent risk factors and those without any SMuRFs 46](#_Toc213598799)

[**Table S22.** Inverse probability weighting: Comparison between patients with both diabetes and hypertension as concurrent risk factors and those without any SMuRFs 48](#_Toc213598800)

[**Table S23. Interaction Test of diabetes-related mortality across additional SMuRF combinations** 50](#_Toc213598801)

# **SUPPLEMENTAL METHODS**

## **The International Survey of Acute Coronary Syndromes (ISACS)** **Archives**.

The ISACS Archives network (NCT04008173) is part of ISACS (NCT01218776) health care program. It is a collaborative network of research centers that support rapid development of new scientific information and analytic tools. The ISACS Archives uses an established informatics infrastructure, hosted and managed by the ISACS TC registry (NCT01218776) and the Department of Electrical and Computer Engineering, University of California, Los Angeles, which enables sharing of data. The ISACS Archives includes sites in which investigators are committed to collecting good-quality data without a strict proportionate sampling. Registries enrolled in the ISACS Archives use data definition for the measures/experiments that are harmonized to the standard variables of the ISACS.^1^ Participation in the research network does not eliminate the ability of any individual patient registry from analyzing only the data from the registry alone.

## **Information on Registries of the ISACS-** **Archives**

Because one of the aims of the current study was to investigate the association between cardiovascular outcomes and time from onset of symptoms to hospital presentation, we identified two large clinical registries providing such information, namely, the ISACS-TC^1^ and the HORACS^2^. Overall, in the current study, the ISACS-Archives collected data from 41 centers in 11 European countries: Bosnia and Herzegovina, Croatia, Italy, Kosovo, Lithuania, Macedonia, Hungary, Moldova, Montenegro, Romania and Serbia. Among these sites, there were 22 tertiary health care services providing percutaneous coronary intervention (PCI). Vital status was available in 100% of participants.

## **Definition of risk factors for CHD**

Smoking habits were self-reported. Persons who were active smokers at time of the index event and smoked regularly during the previous 12 months were classified as current smokers. Former smokers were defined as those patients who had a history of smoking tobacco but were not active smokers in the last 12 months. Hypertension, hypercholesterolemia, and diabetes were assessed by designation of medical history before admission in the database. Family history of coronary artery disease (CAD) was defined as death due to CHD before 55 years of age (for men) and 65 years of age (for women) in any first-degree relative or grandparent. Body mass index (BMI) was calculated as weight (kg) divided by height squared (m^2^). Patients with BMI of 30 or greater were defined as obese.

**Multiple Imputation using Chained Equation (MICE) algorithm.**

Missing data (ranging from 9.8 % to 18.1 % across covariates) were handled using the Multiple Imputation by Chained Equations (MICE) algorithm, an iterative procedure that sequentially imputes each variable with missing values using regression models based on all other variables under the missing-at-random assumption.^3^ To better capture nonlinear relations among predictors and enhance imputation accuracy, we implemented MICE using **XGBoost-based regression models** within each chained equation step.

The variables subjected to imputation correspond to those listed in **Table S1**, including demographic, clinical, laboratory, and treatment characteristics. A total of **ten imputed datasets** were generated to reflect imputation uncertainty. Each dataset was analyzed separately, and the final results were obtained by pooling parameter estimates and standard errors according to **Rubin’s rules.**

Imputation was performed prior to propensity-score estimation, ensuring that inverse probability weighting (IPW) was applied to complete, imputed datasets. This sequence preserves covariate structure and minimizes bias due to missing data. Sensitivity analyses restricted to complete-case observations yielded results consistent with those from the imputed analyses, confirming the robustness of our findings.

**Inverse Propensity Score Weighting Analysis**

Inverse Probability Weighting (IPW) was applied to balance the distribution of baseline covariates between comparison groups. Propensity scores were estimated using logistic regression models including all baseline characteristics listed in Table 1, such as demographic factors, cardiovascular risk factors, history of cardiovascular disease, and clinical presentation variables. Individual weights were defined as:

wi​=ei​Zi​​+1−ei​1−Zi​​

where Z indicates group membership and e the estimated propensity score (P (Z = 1 | x)). Weights were stabilized using the marginal probability of exposure and trimmed at the 1st and 99th percentiles (corresponding to thresholds < 0.01 and > 0.99) to minimize the influence of extreme values.

**Covariate balance** after weighting was evaluated using **standardized mean differences (SMD),** with < 0.10 considered indicative of satisfactory balance. As shown in **Table S3**, all baseline variables met this criterion, confirming adequate post-weighting balance and model stability

To assess robustness, analyses were repeated using conventional multivariable regression models without weighting, and using XGBoost-based propensity estimation as an alternative nonparametric approach. All methods yielded consistent results, confirming the reliability of the IPW-adjusted estimates.

## **Computation of Relative Risk and its Confidence Interval**

All reported risk ratios (RRs) were derived from IPW-adjusted regression models with robust variance estimation in the weighted pseudo-population. In a two-group cohort study, the risk ratio (RR, also called relative risk), is usually applied to compare risks of a health event between two independent binomial populations that differ by a demographic characteristic (i.e. sex, age) or by the level of exposure to a specific risk factor. In such types of studies, data can be summarized in a confusion matrix as follows:

|  | **Risk of Designated Outcome** | |  |
| --- | --- | --- | --- |
|  | **Yes** | **No** | **Total** |
| **Exposed** | a | b | a+b (*H_1_*) |
| **Unexposed** | c | d | c+d (*H_0_*) |
| **Total** | a+c | b+d |  |

Where *H_1_* and *H_0_* correspond to the total number of exposed and unexposed patients, respectively, whereas *a and c* represent the number of exposed and unexposed patients at risk for the designated outcome, respectively.

RR is defined as the ratio between the risk of outcome in exposed patients (*H_1_*) and the risk of outcome in unexposed patients (*H_0,_*) which can be summarized as:

$$RR=\frac{\left( \frac{a}{H_{1}} \right)}{\left( \frac{c}{H_{0}} \right)}$$

When applying this equation to an IPW balanced population, $\frac{a}{H_{1}}$ will be assigned a weight equal to the reciprocal of the propensity score ($\frac{1}{e}$) and $\frac{c}{H_{0}}$ will be weighted by the reciprocal of one minus the propensity score ($\frac{1}{(1-e)}$).

In order to compute the lower and upper (1-α) confidence limit RR_L_ for RR, we operate in the assumption of log normal distribution^5^. In particular, the variate $\log\frac{\left( \frac{a}{H_{1}} \right)}{\left( \frac{c}{H_{0}} \right)}$= $\log\frac{a}{H_{1}}- \log\frac{c}{H_{0}}$is approximately normally distributed with approximate mean log(RR) and estimated variance $\frac{1-\left( \frac{a}{H_{1}} \right)}{a}$ + $\frac{1-(\frac{c}{H_{0}})}{c}$ .

It follows that RR_L_ can be computed by solving the following equation:

$$\frac{\left[ log( \frac{\frac{a}{H_{1}}}{\frac{c}{H_{0}}})- \log({RR}_{L}) \right]}{\left[ \frac{1-\left( \frac{a}{H_{1}} \right)}{a} + \frac{1-(\frac{c}{H_{0}})}{c} \right]^{1/2}}=z_{1-\alpha}$$

Where $z_{1-\alpha}$, is the 100(1-α) percentage point of the N(O, 1) distribution

## **Comparison of means and prevalences in the weighted sample**

To evaluate the balance of the baseline covariate distributions between treatment and control groups, standardized difference (SD) is widely used in inverse probability weighting (IPW) framework. For the baseline analysis, we use standard SD which is defined as follows: $\frac{m_{t}-m_{c}}{\sqrt{\frac{s_{t}^{2}+s_{c}^{2}}{2}}}$ for continuous variables and $\frac{m_{t}-m_{c}}{\sqrt{\frac{m_{t}(1-m_{t})+m_{c}(1-m_{c})}{2}}}$ for binary variables where $m_{t}, m_{c}$ are sample mean of the variables for treatment and control group, and $s_{t}^{2}, s_{c}^{2}$ are sample variance of the variables for treatment and control group, respectively. For IPW analysis, we use weighted SD where $m_{t}, m_{c}$ are replaced with weighted sample mean of the variables for treatment and control group, and $s_{t}^{2}, s_{c}^{2}$ are replaced with weighted sample variance of the variables for treatment and control group, respectively. Weights are determined by the inverse probability of treatment received. In general, 0.1 is the reasonable threshold to determine whether two distributions are balanced (i.e., if SD >0.1, the baseline covariate is imbalanced).^6^

**Interaction test**

The comparison of two estimated quantities, each with its standard error, is a general method that can be applied widely. Outcome measures were analyzed on the log scale because the distributions of the log ratios tend to be closer to normal than of the ratios themselves. If the estimates are *E*1 and *E*2 with standard errors SE(*E*1) and SE(*E*2), then the difference *d*=*E*1 - *E*2 has standard error SE(*d*)=Ö[SE(*E*1)2 + SE(*E*2)2] i.e., the square root of the sum of the squares of the separate standard errors. The ratio *z*=*d*/SE(*d*) gives a test of the null hypothesis that in the population the difference *d* is zero, by comparing the value of *z* to the standard normal distribution. The 95% confidence interval (CI) for the difference is *d*-1.96SE(*d*) to *d*+1.96SE(*d*).^7^

**Interaction and trend analyses.**

To assess whether the mortality risk associated with diabetes was attenuated in the presence of additional SMuRFs, formal interaction testing was performed using the method proposed by Altman DG and Bland JM ^7^. For each sex, RRs for 30-day mortality were estimated from IPW-adjusted regression models for diabetes alone and for diabetes coexisting with individual SMuRFs (hypertension, hypercholesterolemia, or current smoking), using the SMuRF-less group as reference. Interaction was tested by comparing log-transformed RRs and their standard errors across subgroups. As mentioned above, the z-statistic was computed as the ratio of the difference between two log-RRs (d = E₁ – E₂) to its standard error (SE(d) = √[SE(E₁)² + SE(E₂)²]), assuming normality of the log-scale estimates. One-sided p-values < 0.05 were considered statistically significant.

# **SUPPLEMENTAL RESULTS**

## **Figure S1.** Study flow chart


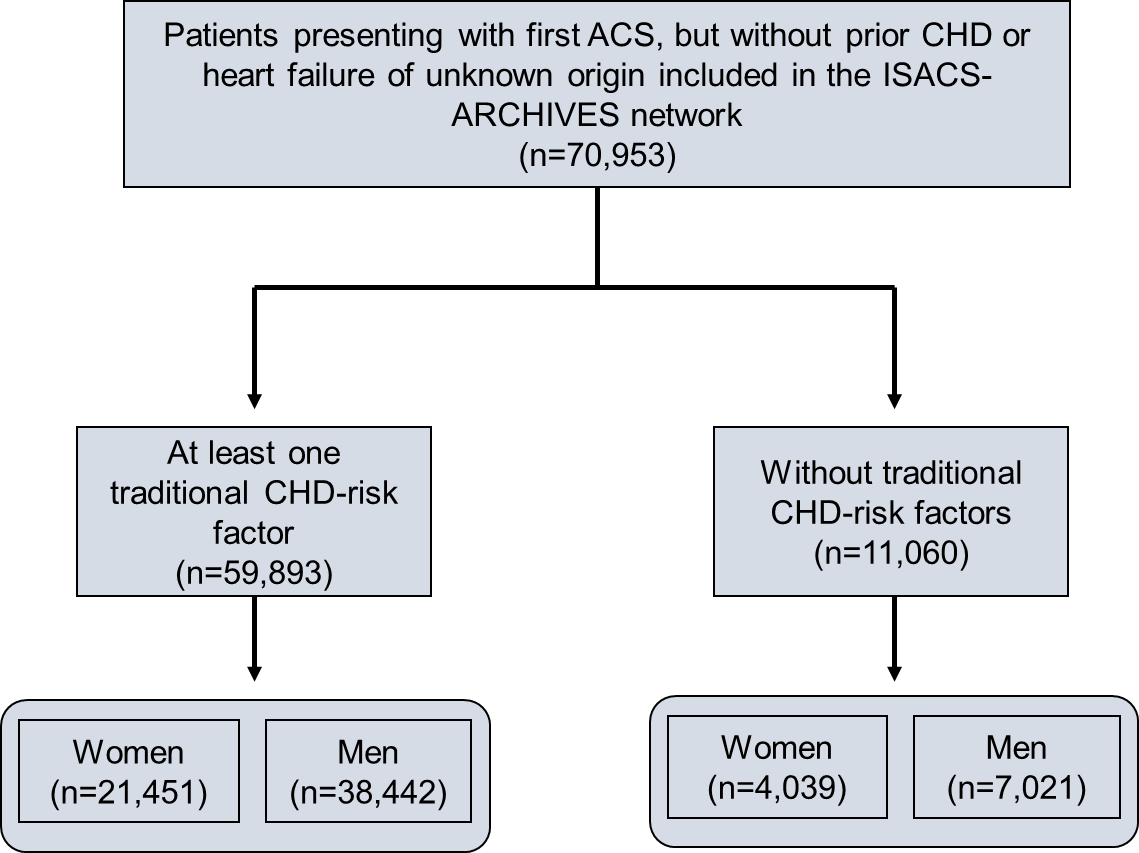


**Abbreviations:** ACS, acute coronary syndromes; CHD, coronary heart disease; NSTE-ACS, non-ST-segment elevation acute coronary syndromes; STEMI, ST-segment elevation acute coronary syndromes

## **Figure S2.** Prevalence of SMuRF among men and women hospitalized for acute coronary syndromes


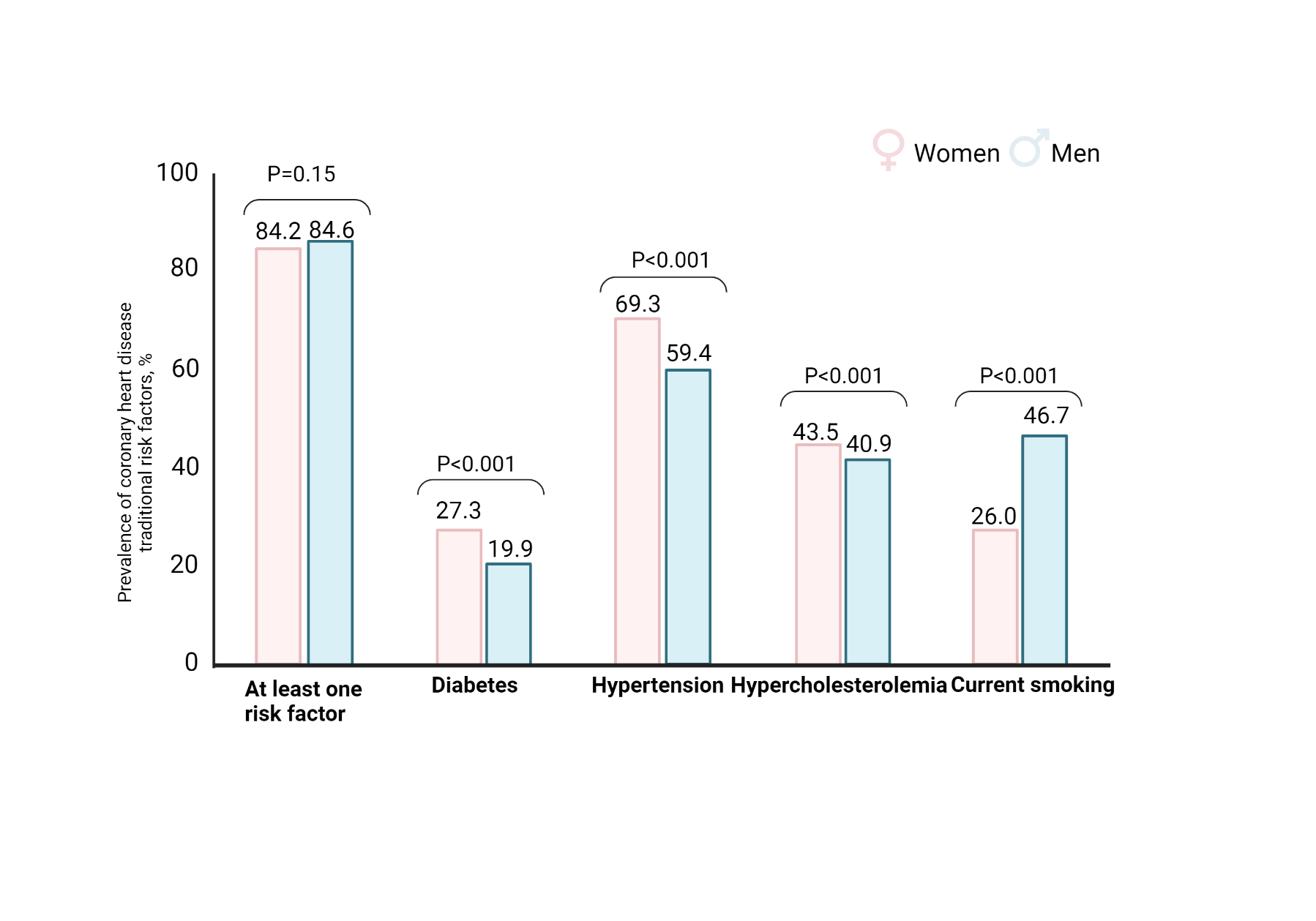


| **Table S1. Baseline characteristics** | | | | |
| --- | --- | --- | --- | --- |
|  | **SMuRFs**  **N= 59,893** | **SMuRF- less N=11,060** | **Standardized difference** | |
| Age (years), mean ± SD | 62.6 ± 11.8 | 64.4 ± 12.3 | -0.15 |  |
| Women, % | 21,451 (35.8) | 4,039 (36.5) | -0.01 |  |
| **Coronary heart disease risk factors** | | | | |
| Diabetes, % | 15,988 (26.7) | 0.0 (0.0) | 0.85 |  |
| Hypertension, % | 44,678 (74.6) | 0.0 (0.0) | 2.42 |  |
| Hypercholesterolemia, % | 29,658 (49.5) | 0.0 (0.0) | 1.40 |  |
| Current smokers, % | 27,860 (46.5) | 0.0 (0.0) | 1.32 |  |
| Former smokers, % | 1,068 (1.8) | 184 (1.7) | 0.009 |  |
| Family history of CAD, % | 19,389 (32.4) | 1,609 (14.5) | 0.43 |  |
| BMI ≥30 kg/m^2^, % | 12,592 (21.0) | 1,446 (13.1) | 0.21 |  |
| **Clinical history of cardiovascular disorders** | | | | |
| Prior stroke, % | 2,415 (4.0) | 327 (3.0) | 0.06 |  |
| Peripheral artery disease, % | 1,526 (2.5) | 129 (1.2) | 0.10 |  |
| **Clinical history of comorbidities** | | | | |
| Chronic kidney disease, % | 2,897 (4.8) | 387 (3.5) | 0.07 |  |
| **Clinical presentation** | | | | |
| STEMI, % | 37,512 (62.6) | 5,917 (53.5) | 0.19 |  |
| NSTEMI, % | 11,952 (20.0) | 1,581 (14.3) | 0.15 |  |
| Unstable angina, % | 10,429 (17.4) | 3,562 (32.2) | -0.35 |  |
| NSTE-ACS, % | 22,381 (37.4) | 5,143 (46.5) | -0.19 |  |
| ST-segment shifts in anterior leads (at ECG) ,% | 12,989 (21.7) | 2,074 (18.8) | 0.07 |  |
| Time to admission <12 hours, % | 44,741 (74.7) | 7,895 (71.4) | 0.07 |  |
| Time to admission <2 hours, % | 13,508 (22.6) | 2,238 (20.2) | 0.06 |  |
| HR at admission (bpm), mean ± SD | 81.9 ± 19.9 | 80.4 ± 19.6 | 0.08 |  |
| SBP at admission (mmHg), mean ± SD | 139.2 ± 28.2 | 131.1 ± 29.5 | 0.28 |  |
| **Revascularization therapy for STEMI** | | |  |  |
| Reperfusion therapy (fibrinolysis, PCI or CABG), % | 25,731 (43.0) | 3,648 (33.0) | 0.21 |  |
| PCI, % | 16,456 (27.5) | 2,311 (20.9) | 0.15 |  |
| Fibrinolysis, % | 11,113 (18.6) | 1,548 (14.0) | 0.12 |  |
| Fibrinolysis and PCI, % | 1,958 (3.3) | 233 (2.1) | 0.07 |  |
| CABG, % | 765 (1.3) | 134 (1.2) | 0.01 |  |
| **Invasive cardiac procedures** | | | | |
| PCI (all ACS), % | 22,163 (37.0) | 3,266 (29.5%) | 0.16 |  |
| CABG (all ACS), % | 1,301 (2.2) | 197 (1.8) | 0.03 |  |
| **Medications on admission** | | | | |
| Aspirin and/or P2Y_12_ inhibitors, % | 56,167 (93.8) | 8,499 (76.8) | 0.49 |  |
| Unfractionated heparin, % | 33,401 (55.8) | 6,121 (55.3) | 0.01 |  |
| LMWH, % | 27,770 (46.4) | 3,215 (29.1) | 0.36 |  |
| Heparins (all), % | 52,477 (87.6) | 7,890 (71.3) | 0.41 |  |
| GP IIb/IIIa inhibitors, % | 4,284 (7.2) | 754 (6.8) | 0.01 |  |
| **Medications during** **hospitalization and at discharge** | | | | |
| β-blockers, % | 40,714 (68.0) | 5,268 (47.6) | 0.42 |  |
| ACE-inhibitors/ARBs, % | 43,482 (72.6) | 5,185 (46.9) | 0.54 |  |
| Statins, % | 47,224 (78.8) | 6,616 (59.8) | 0.42 |  |
| **Outcomes** |  |  | **P value** | |
| 30-day mortality | 4,631 (7.7) | 1,466 (13.3) | <0.0001 | |
| Values are number (percentage) or mean ± SD unless stated otherwise.  **Abbreviations:** ACE=angiotensin converting enzyme; ARBs=angiotensin receptor blockers; BMI=body mass index; bpm = beats per minute; CABG=coronary artery bypass graft; CAD=coronary artery disease; CHD = coronary heart disease; ECG=electrocardiogram; GP=glycoprotein, HR=heart rate; LMWH=low molecular weight heparins; NSTE-ACS=non-ST-segment elevation acute coronary syndrome; NSTEMI=ST-segment elevation myocardial infarction; PCI=percutaneous coronary intervention; SBP=systolic blood pressure; SMuRF: standard modifiable cardiovascular risk factor; STEMI=ST-segment elevation myocardial infarction | | | | |

| **Table S2.** Interaction test: calculations for comparing two estimated risk ratios (women vs men) for 30-day mortality by inverse probability weighting: SMuRFs versus SMuRFs less patients | | | |
| --- | --- | --- | --- |
|  |  | **Group 1**  **[Women]**  **(n = 25,490)** | **Group 2**  **[Men]**  **(n = 45,463)** |
| **1** | **RR** | 0.72 | 0.64 |
| **2** | **log RR** | - 0.32 | - 0.44 |
| **3** | **95% CI for RR** | (0.65 – 0.79) | (0.59 – 0.70) |
| **4** | **95% CI for log RR** | -0.43 – (-0.23) | -0.52 – (-0.35) |
| **5** | **Width of CI** | 0.19 | 0.17 |
| **6** | **SE (=width / (2*1.96))** | 0.05 | 0.04 |
|  | | | |
| **7** | **d (=**$\boldsymbol{E}_{\boldsymbol{1}}\boldsymbol{-}\boldsymbol{E}_{\boldsymbol{2}}$**)** | **0.12** | |
| **8** | **SE (d)** | **0.07** | |
| **9** | **CI (d)** | -0.01 – 0.24 | |
| **10** | **Test of Interaction** | 1.78 (***P* value=0.04**) | |
|  | | | |
| **11** | **RRR (=exp(d) )** | 1.12 | |
| **12** | **CI (RRR)** | 0.99 – 1.28 | |

| **Table S3.** Inverse probability weighting: clinical factors and outcomes stratified by SMuRFs status | | | |
| --- | --- | --- | --- |
| **Characteristics** | **SMuRFs**  **(N=59,893)** | **SMuRF-less (N=11,060)** | **Standardized difference** |
| Age (years), mean ± SD | 62.9 ± 11.8 | 62.6 ± 12.7 | 0.0218 |
| **Coronary heart disease risk factors** | |  |  |
| Family history of CAD | 29.6 | 29.6 | -0.0005 |
| Former smokers | 1.8 | 1.9 | -0.0115 |
| BMI ≥30 kg/m^2^ | 19.8 | 20.0 | -0.0062 |
| **Clinical history of cardiovascular disorders** |  |  |  |
| Peripheral artery disease | 2.3 | 2.1 | 0.0126 |
| Prior stroke | 3.9 | 4.0 | -0.0069 |
| **Clinical presentation on admission** | |  |  |
| ST-segment shifts in anterior leads (at ECG) | 21.2 | 21.4 | -0.0044 |
| SBP at admission (mmHg), mean ± SD | 137.9 ± 28.4 | 138.4 ± 29.9 | -0.0167 |
| HR at admission (bpm), mean ± SD | 81.7 ± 19.9 | 81.8 ± 20.2 | -0.0041 |
| **Outcomes** |  |  | **P value** |
| 30-day mortality | 8.1 | 11.4 |  |
| Risk Ratio (95% CI) | 0.68 (0.64 to 0.73) | | <0.0001 |
| Values are percentage or mean ± SD unless stated otherwise.  **Abbreviations:** BMI=body mass index; bpm = beats per minute; CAD=coronary artery disease; CHD= coronary heart disease; ECG=electrocardiogram; HR=heart rate; SBP= systolic blood pressure; SMuRF: standard modifiable cardiovascular risk factor; | | | |

| **Table S4.** Inverse probability weighting: clinical factors and outcomes stratified by SMuRFs status in patients undergoing reperfusion therapy for **STEMI: PCI, fibrinolysis, or CABG**. | | | |
| --- | --- | --- | --- |
| **Characteristics** | **SMuRFs (N=25,731)** | **SMuRF-less (N=3,648)** | **Standardized difference** |
| Age, years Mean ± SD | 60.7 ± 11.6 | 60.2 ± 12.6 | 0.0375 |
| **Coronary heart disease risk factors** | | | |
| Family history of CAD ,% | 28.0 | 28.1 | -0.0030 |
| Former smokers,% | 2.4 | 2.8 | -0.0206 |
| BMI ≥30 kg/m^2^,% | 20.6 | 21.1 | -0.0111 |
| **Clinical history of CVD** |  |  |  |
| PAD,% | 1.7 | 1.8 | -0.0033 |
| Prior stroke,% | 3.0 | 3.1 | -0.0052 |
| **Clinical presentation on admission** | |  |  |
| ST-segment shifts in anterior leads (at ECG) ,% | 28.4 | 27.7 | 0.0155 |
| SBP at admission, mmHg Mean ± SD | 137.4 ± 28.0 | 137.7 ± 28.7 | -0.0115 |
| HR at admission, bpm Mean ± SD | 80.6 ± 19.2 | 80.6 ± 18.6 | -0.0021 |
| **Outcomes** |  |  | **P value** |
| 30-day mortality | 7.0 | 10.8 |  |
| Risk Ratio (95% CI) | 0.62 (0.55 – 0.70) | | <0.0001 |
| Values are percentages (%) or mean ±standard deviation, unless otherwise specified.  **Abbreviations**: BMI, body mass index; bpm, beats per minute; CABG, coronary artery bypass graft; CAD, coronary artery disease; CHD, coronary heart disease; CVD, cardiovascular disorders; ECG, electrocardiogram; HR, heart rate; PAD, peripheral artery disease; PCI, percutaneous coronary intervention; SBP, systolic blood pressure; SMuRF: standard modifiable cardiovascular risk factor; STEMI, ST-segment elevation myocardial infarction. | | | |

| **Table S5.** Inverse probability of weighting: outcomes stratified by SMuRFs status and time from symptom onset to hospital admission in patients undergoing reperfusion therapy for **STEMI (**PCI, fibrinolysis or CABG) | | | | | | | | |
| --- | --- | --- | --- | --- | --- | --- | --- | --- |
|  | **Time to admission≤2 hrs** | | | | . **Time to admission** >**2 hrs** | | | |
| **Characteristics** | **SMuRFs**  **(N=7,374)** | **SMuRF-less**  **(N=963)** | **Standardized difference** | | **SMuRFs**  **(N=18,357)** | **SMuRF- less**  **(N=2,685)** | **Standardized difference** | |
| Age (years) Mean ± SD | 58.6 ± 11.4 | 58.3 ± 12.4 | 0.02 |  | 61.5 ± 11.6 | 61.0 ± 11.8 | 0.02 |  |
| **Cardiovascular risk factors** | | | | | | | | |
| Family history of CAD,% | 34.5 | 34.9 | -0.01 |  | 25.4 | 25.4 | 0.001 |  |
| Former smokers,% | 2.0 | 2.5 | -0.04 |  | 2.6 | 2.8 | -0.01 |  |
| Obesity (BMI≥30),% | 20.7 | 20.8 | -0.002 |  | 20.6 | 21.4 | -0.02 |  |
| **Clinical history of CVD** | | | | | | | | |
| PAD,% | 1.7 | 1.6 | 0.01 |  | 1.7 | 1.9 | -0.01 |  |
| Prior stroke,% | 2.8 | 2.6 | 0.01 |  | 3.1 | 3.4 | -0.01 |  |
| **Clinical presentation on admission** | | | | | | | | |
| ST-segment shifts in anterior leads (at ECG) ,% | 30.4 | 29.4 | 0.02 |  | 26.0 | 25.4 | 0.01 |  |
| SBP at admission (mmHg) Mean ± SD | 136.9 ± 27.8 | 137.2 ± 28.2 | -0.01 |  | 138.2±28.1 | 138.3±29.1 | -0.01 |  |
| HR at admission (bpm) Mean ± SD | 78.9 ± 18.6 | 79.3 ± 18.1 | -0.02 |  | 80.9±19.3 | 80.8±18.8 | 0.01 |  |
| **Outcomes** |  |  | ***P* value** |  |  |  | ***P* value** |  |
| 30-day mortality | 5.5 | 10.4 |  |  | 7.6 | 11.1 |  |  |
| Risk Ratio (95% CI) | 0.50 (0.40 – 0.63) | | <0.0001 |  | 0.66 (0.58 – 0.76) | | <0.0001 |  |
| Values are percentages (%) or mean ±standard deviation, unless otherwise specified.  **Abbreviations**: BMI, body mass index; bpm, beats per minute; CABG, coronary artery bypass graft; CAD, coronary artery disease; CHD, coronary heart disease; CVD, cardiovascular disorders; ECG, electrocardiogram; HR, heart rate; PAD, peripheral artery disease; PCI, percutaneous coronary intervention; SBP, systolic blood pressure; SMuRF: standard modifiable cardiovascular risk factor; STEMI, ST-segment elevation myocardial infarction. | | | | | | | | |

| **Table S6.** Inverse probability weighting: outcomes stratified by SMuRFsstatus in patients undergoing **revascularization therapy (PCI or CABG) in NSTE-ACS** | | | |
| --- | --- | --- | --- |
| **Characteristics** | **SMuRFs**  **(N=6,550)** | **SMuRF-less**  **(N=1,088)** | **Standardized difference** |
| Age, years Mean ± SD | 60.7 ± 11.4 | 60.5 ± 12.1 | 0.02 |
| **Coronary heart disease risk factors** | | | |
| Family history of CAD ,% | 24.7 | 24.8 | -0.004 |
| Former smokers,% | 2.9 | 3.5 | -0.04 |
| BMI ≥30 kg/m^2^,% | 23.4 | 23.5 | -0.002 |
| **Clinical history of CVD** |  |  |  |
| PAD,% | 1.6 | 1.1 | 0.04 |
| Prior stroke ,% | 3.0 | 2.4 | 0.04 |
| **Clinical presentation on admission** | | | |
| ST-segment shifts in anterior leads (at ECG) ,% | 10.8 | 10.9 | -0.005 |
| SBP at admission, mmHg Mean ± SD | 144.8 ± 26.5 | 145.4 ± 28.6 | -0.02 |
| HR at admission, bpm Mean ± SD | 80.5 ± 18.4 | 80.1 ± 17.8 | 0.02 |
| **Outcomes** |  |  | **P value** |
| 30-day mortality | 2.6 | 3.8 |  |
| Risk Ratio (95% CI) | 0.68 (0.48 – 0.97) | | 0.03 |
| Values are number (percentage) or mean ± SD unless stated otherwise.  **Abbreviations:** BMI=body mass index; bpm = beats per minute; CABG=coronary artery bypass graft; CAD=coronary artery disease; CHD = coronary heart disease; ECG=electrocardiogram; HR=heart rate; NSTE-ACS=non-ST-segment elevation acute coronary syndrome; PCI=percutaneous coronary intervention; SBP=systolic blood pressure; SMuRF: standard modifiable cardiovascular risk factor; | | | |

| **Table S7.** Inverse probability weighting: outcomes stratified by SMuRFs status in **aspirin and/or P2Y_12_ inhibitors users** | | | |
| --- | --- | --- | --- |
| **Characteristics** | **SMuRFs**  **(N=56,167)** | **SMuRF-less**  **(N=8,499)** | **Standardized difference** |
| Age, years Mean ± SD | 62.8 ± 11.8 | 62.5 ± 12.8 | 0.01 |
| **Coronary heart disease risk factors** |  |  |  |
| Family history of CAD ,% | 30.5 | 30.3 | 0.01 |
| Former smokers,% | 1.9 | 2.1 | -0.01 |
| BMI ≥30 kg/m^2^,% | 20.0 | 20.0 | -0.0003 |
| **Clinical history of CVD** |  |  |  |
| PAD ,% | 2.4 | 2.2 | 0.02 |
| Prior stroke ,% | 3.9 | 3.8 | 0.01 |
| **Clinical presentation on admission** |  |  |  |
| ST-segment shifts in anterior leads (at ECG) ,% | 21.6 | 21.8 | -0.01 |
| SBP at admission, mmHg Mean ± SD | 139.3 ± 28.0 | 139.0 ± 28.4 | 0.01 |
| HR at admission, bpm Mean ± SD | 81.6 ± 19.8 | 81.7±19.8 | -0.01 |
| **Medications on admission** |  |  |  |
| GP IIb/IIIa inhibitors ,% | 7.1 | 7.0 | 0.003 |
| Heparins (all) ,% | 89.5 | 89.1 | 0.01 |
| **Medications during hospitalization and at discharge** | | | |
| ACE inhibitors/ARBs ,% | 72.3 | 72.1 | 0.003 |
| Statins,% | 81.1 | 81.0 | 0.002 |
| β-blockers,% | 68.3 | 68.7 | -0.01 |
| **Outcomes** |  |  | **P value** |
| 30-day mortality | 7.4 | 8.7 |  |
| Risk Ratio (95% CI) | 0.84 (0.77 – 0.91) | | 0.0001 |
| Values are number (percentage) or mean ± SD unless stated otherwise.  **Abbreviations:** ACE=angiotensin converting enzyme; ARBs=angiotensin receptor blockers; BMI=body mass index; bpm = beats per minute; CAD=coronary artery disease; CHD = coronary heart disease; ECG=electrocardiogram; GP=glycoprotein, HR=heart rate; SBP=systolic blood pressure; SMuRF: standard modifiable cardiovascular risk factor; | | | |

| **Table S8.** Inverse probability weighting: outcomes stratified by SMuRFs status in **heparin users** | | | |
| --- | --- | --- | --- |
| **Characteristics** | **SMuRFs**  **(N=52,477)** | **SMuRF-less**  **(N=7,890)** | **Standardized difference** |
| Age, years Mean ± SD | 62.7 ± 11.8 | 62.5 ± 12.8 | 0.02 |
| **Coronary heart disease risk factors** | | | |
| Family history of CAD ,% | 30.2 | 30.2 | -0.0004 |
| Former smokers,% | 1.8 | 1.9 | -0.01 |
| BMI ≥30 kg/m^2^,% | 20.0 | 20.2 | -0.01 |
| **Clinical history of CVD** |  |  |  |
| PAD ,% | 2.4 | 2.1 | 0.02 |
| Prior stroke ,% | 3.9 | 3.8 | 0.01 |
| **Clinical presentation on admission** |  |  |  |
| ST-segment shifts in anterior leads (at ECG) ,% | 20.8 | 21.2 | -0.01 |
| SBP at admission, mmHg Mean ± SD | 140.1 ± 27.8 | 139.9 ± 28.4 | 0.01 |
| HR at admission, bpm Mean ± SD | 81.8 ± 19.9 | 81.8 ± 19.6 | 0.0001 |
| **Medications on admission** |  |  |  |
| GP IIb/IIIa inhibitors,% | 6.3 | 6.1 | 0.01 |
| Aspirin/P2Y_12_ inhibitors,% | 95.9 | 96.0 | -0.003 |
| **Medications during hospitalization and at discharge** | | | |
| ACE inhibitors/ARBs ,% | 72.7 | 72.8 | -0.002 |
| Statins,% | 80.5 | 80.5 | 0.0002 |
| β-blockers ,% | 68.7 | 69.4 | -0.01 |
| **Outcomes** |  |  | **P value** |
| 30-day mortality | 7.6 | 9.0 |  |
| Risk Ratio (95% CI) | 0.83 (0.77 – 0.90) | | <0.0001 |
| Values are number (percentage) or mean ± SD unless stated otherwise.  **Abbreviations:** ACE=angiotensin converting enzyme; ARBs=angiotensin receptor blockers; BMI=body mass index; bpm = beats per minute; CAD=coronary artery disease; CHD = coronary heart disease; ECG=electrocardiogram; GP=glycoprotein, HR=heart rate; SBP=systolic blood pressure; SMuRF: standard modifiable cardiovascular risk factor; | | | |

| **Table S9**. Inverse probability weighting: outcomes stratified by SMuRFs status in Glycoprotein IIb/IIIa inhibitors users | | | |
| --- | --- | --- | --- |
| **Characteristics** | **SMuRFs (N=4,284)** | **SMuRF- less (N=754)** | **Standardized difference** |
| Age, years Mean ± SD | 61.9 ± 11.9 | 61.9 ± 13.3 | 0.002 |
| **Coronary heart disease risk factors** | | | |
| Family history of CAD,% | 28.9 | 26.3 | 0.06 |
| Former smokers,% | 3.5 | 4.1 | -0.03 |
| BMI ≥30 kg/m^2^,% | 23.2 | 23.9 | -0.02 |
| **Clinical history of CVD** |  |  |  |
| PAD,% | 1.7 | 2.9 | -0.06 |
| Prior stroke,% | 2.9 | 2.2 | 0.04 |
| **Clinical presentation on admission** |  |  |  |
| ST-segment shifts in anterior leads (at ECG),% | 34.8 | 33.8 | 0.02 |
| SBP at admission, mmHg Mean ± SD | 132.9 ± 28.5 | 132.8 ± 29.1 | 0.01 |
| HR at admission, bpm Mean ± SD | 81.1 ± 19.6 | 81.3 ± 20.5 | -0.01 |
| **Medications on admission** |  |  |  |
| Heparins (all),% | 75.7 | 76.4 | -0.02 |
| Aspirin/ P2Y12 inhibitors ,% | 91.3 | 91.7 | -0.02 |
| **Medications during hospitalization and at discharge** | | | |
| ACE inhibitors/ARBs,% | 67.1 | 66.8 | 0.01 |
| Statins,% | 74.6 | 75.8 | -0.03 |
| β-blockers,% | 62.2 | 63.2 | -0.02 |
| **Outcomes** |  |  | **P value** |
| 30-day mortality | 8.7 | 9.0 |  |
| Risk Ratio (95% CI) | 0.96 (0.73 – 1.26) | | 0.77 |
| Values are number (percentage) or mean ± SD unless stated otherwise.  **Abbreviations:** ACE=angiotensin converting enzyme; ARBs=angiotensin receptor blockers; BMI=body mass index; bpm = beats per minute; CAD=coronary artery disease; CHD = coronary heart disease; ECG=electrocardiogram; GP=glycoprotein, HR=heart rate; SBP=systolic blood pressure | | | |

| **Table S10.** **Interaction test**: calculations for comparing two estimated risk ratios (women vs men) for 30-day mortality by inverse probability weighting: diabetes as their sole SMuRF versus SMuRF-less. | | | |
| --- | --- | --- | --- |
|  |  | **Group 1**  **[Women]**  **(n = 25,490)** | **Group 2**  **[Men]**  **(n = 45,463)** |
| **1** | **RR** | 1.29 | 1.40 |
| **2** | **log RR** | 0.25 | - 0.44 |
| **3** | **95% CI for RR** | (1.06 – 1.57) | (1.16 – 1.69) |
| **4** | **95% CI for log RR** | 0.06-0.45 | 0.15-0.52 |
| **5** | **Width of CI** | 0.39 | 0.38 |
| **6** | **SE (=width / (2*1.96))** | 0.10 | 0.10 |
|  | | | |
| **7** | **d (=**$\boldsymbol{E}_{\boldsymbol{1}}\boldsymbol{-}\boldsymbol{E}_{\boldsymbol{2}}$**)** | **-0.08** | |
| **8** | **SE (d)** | **0.14** | |
| **9** | **CI (d)** | -0.35-0.19 | |
| **10** | **Test of Interaction** | -0.59 (***P* value=0.28**) | |
|  | | | |
| **11** | **RRR (=exp(d) )** | 0.92 | |
| **12** | **CI (RRR)** | 0.70-1.21 | |

| **Table S11.** Inverse probability weighting: outcomes stratified by SMURFS status. Comparison between patients with diabetes as a solitary risk factor and those without any SMuRFs in the subgroup undergoing reperfusion therapy for **STEMI (**PCI, fibrinolysis, or CABG). | | | | |
| --- | --- | --- | --- | --- |
| **Characteristics** | **Diabetes (N=1162)** | **SMuRF-less**  **(N=5917)** | **Standardized difference** | |
| Age, years Mean ± SD | 65.9±11.2 | 65.6±12.6 | 0.03 |  |
| **Cardiovascular risk factors** | | | | |
| Family history of CAD, % | 15.2 | 15.1 | 0.001 |  |
| Former smokers, % | 2.3 | 2.3 | -0.001 |  |
| BMI ≥30 kg/m^2^, % | 12.5 | 12.7 | -0.01 |  |
| **Clinical history of CVD** | | | | |
| PAD, % | 1.6 | 1.6 | 0.001 |  |
| Prior stroke, % | 3.5 | 3.5 | 0.002 |  |
| **Clinical presentation on admission** | | | | |
| ST-segment shifts in anterior leads (at ECG), % | 27.8 | 27.2 | 0.01 |  |
| SBP at admission, mmHg Mean ± SD | 128.1±30.8 | 128.3±30.2 | -0.01 |  |
| HR at admission, bpm Mean ± SD | 81.9±20.6 | 81.9±20.7 | 0.002 |  |
| **Outcomes** | | | ***P* value** |  |
| 30-day mortality, % | 22.6 | 20.0 |  |  |
| Risk Ratio (95% CI) | 1.16 (1.00 – 1.35) | | 0.0517 |  |
| Data are presented as percentages (%) or mean ±standard deviation, unless otherwise specified.  Abbreviations: BMI=body mass index; CAD=coronary artery disease, CHD=coronary heart disease, CVD=cardiovascular disorders, ECG=electrocardiogram, HR=heart rate; PAD=peripheral artery disease, SBP=systolic blood pressure; SMuRF: standard modifiable cardiovascular risk factor; STEMI, ST-segment elevation myocardial infarction | | | | |

| **Table S12**. Inverse probability weighting: Patients undergoing reperfusion therapy for **STEMI** (PCI, fibrinolysis, or CABG) at different times from symptom onset to hospital admission (≤2 hours vs **>** hours). Comparison between patients with diabetes as a solitary risk factor and those without any SMuRFs | | | | | | | | |
| --- | --- | --- | --- | --- | --- | --- | --- | --- |
|  | **Time to admission ≤2 hours** | | | | **. Time to admission >2 hours** | | | |
| **Characteristics** | **Diabetes (N=199)** | **SMuRF-less**  **(N=1246)** | **Standardized difference** | | **Diabetes (N=963)** | **SMuRF-less**  **(N=4671)** | **Standardized difference** | |
| Age, years Mean ± SD | 63.3±11.5 | 62.7±12.7 | 0.05 |  | 66.6±11.1 | 66.3±12.4 | 0.02 |  |
| **Cardiovascular risk factors** | | | | |  |  |  |  |
| Family history of CAD, % | 21.0 | 20.1 | 0.02 |  | 13.8 | 13.8 | -0.001 |  |
| Former smokers, % | 2.0 | 2.3 | -0.03 |  | 2.4 | 2.3 | 0.004 |  |
| BMI ≥30 kg/m^2^, % | 12.4 | 12.8 | -0.01 |  | 12.4 | 12.7 | -0.01 |  |
| **Clinical history of CVD** | | | | |  |  |  |  |
| PAD, % | 1.7 | 1.7 | 0.0003 |  | 1.6 | 1.6 | 0.0003 |  |
| Prior stroke, % | 4.4 | 4.0 | 0.02 |  | 3.3 | 3.4 | -0.001 |  |
| **Clinical presentation on admission** | | | | |  |  |  |  |
| ST-segment shifts in anterior leads (at ECG), % | 33.2 | 31.7 | 0.03 |  | 26.3 | 26.1 | 0.01 |  |
| SBP at admission, mmHg Mean ± SD | 129.7±29.9 | 130.3±29.5 | -0.02 |  | 127.7±30.9 | 127.8±30.3 | -0.01 |  |
| HR at admission, bpm Mean ± SD | 79.7±17.6 | 79.3±19.8 | 0.02 |  | 82.5±21.1 | 82.5±20.9 | -0.001 |  |
| **Outcomes** | | | ***P* value** |  |  |  | ***P* value** |  |
| 30-day mortality, % | 24.1 | 16.9 |  |  | 22.3 | 20.9 |  |  |
| Risk Ratio (95% CI) | 1.56 (1.09 – 2.24) | | 0.01 |  | 1.09 (0.92 – 1.29) | | 0.3095 |  |
| Data are presented as percentages (%) or mean ±standard deviation, unless otherwise specified.  Abbreviations: BMI=body mass index; CAD=coronary artery disease, CHD=coronary heart disease, CVD=cardiovascular disorders, ECG=electrocardiogram, HR=heart rate; PAD=peripheral artery disease, SBP=systolic blood pressure; SMuRF: standard modifiable cardiovascular risk factor; STEMI, ST-segment elevation myocardial infarction | | | | | | | | |

| **Table S13.** Inverse probability weighting: Patients undergoing reperfusion therapy for **NSTE-ACS** (PCI, fibrinolysis, or CABG). Outcomes stratified by SMURFS status. Comparison between patients with diabetes as a solitary risk factor and those without any SMuRFs | | | | |
| --- | --- | --- | --- | --- |
| **Characteristics** | **Diabetes (N=529)** | **SMuRF-less**  **(N=5143)** | **Standardized difference** | |
| Age, years Mean ± SD | 64.2±10.9 | 63.7±11.9 | 0.04 |  |
| **Cardiovascular risk factors** | | | | |
| Family history of CAD, % | 13.7 | 14.1 | -0.01 |  |
| Former smokers, % | 0.8 | 0.7 | 0.004 |  |
| BMI ≥30 kg/m2, % | 14.7 | 13.9 | 0.02 |  |
| **Clinical history of CVD** | | | | |
| PAD, % | 1.3 | 1.3 | 0.006 |  |
| Prior stroke, % | 2.4 | 2.7 | -0.02 |  |
| **Clinical presentation on admission** | | | | |
| ST-segment shifts in anterior leads (at ECG), % | 9.5 | 9.8 | -0.01 |  |
| SBP at admission, mmHg Mean ± SD | 134.9±25.6 | 134.2±28.7 | 0.02 |  |
| HR at admission, bpm Mean ± SD | 79.5±20.7 | 79.7±19.2 | -0.01 |  |
| **Outcomes** |  | | ***P* value** |  |
| 30-day mortality, % | 9.1 | 6.3 |  |  |
| Risk Ratio (95% CI) | 1.49 (1.08 – 2.04) | | 0.01 |  |
| Data are presented as percentages (%) or mean ±standard deviation, unless otherwise specified.  Abbreviations: BMI=body mass index; CABG=coronary artery bypass graft, CAD=coronary artery disease, CHD=coronary heart disease, CVD=cardiovascular disorders, ECG=electrocardiogram, HR=heart rate; NSTE-ACS= Non-St elevation Acute Coronary Syndrome; PAD=peripheral artery disease, PCI=percutaneous coronary intervention, SBP=systolic blood pressure; SMuRF: standard modifiable cardiovascular risk factor; | | | | |

| **Table S14.** Inverse probability weighting: and outcomes sorted by SMURFS status. Comparison between patients with diabetes as a solitary risk factor and those without any SMuRFs in the subgroup undergoing heparins’use. | | | | |
| --- | --- | --- | --- | --- |
| **Characteristics** | **Diabetes (N=1438)** | **SMuRF-less**  **(N=7890)** | **Standardized difference** | |
| Age, years Mean ± SD | 65.4±11.0 | 64.8±12.4 | 0.05 |  |
| **Cardiovascular risk factors** | | | | |
| Family history of CAD, % | 14.9 | 14.8 | 0.001 |  |
| Former smokers, % | 2.0 | 2.0 | <0.001 |  |
| BMI ≥30 kg/m2, % | 13.3 | 13.4 | -0.004 |  |
| **Clinical history of CVD** | | | | |
| PAD, % | 1.7 | 1.6 | 0.004 |  |
| Prior stroke, % | 3.3 | 3.4 | -0.01 |  |
| **Clinical presentation on admission** | | | | |
| ST-segment shifts in anterior leads (at ECG), % | 19.2 | 18.8 | 0.01 |  |
| SBP at admission, mmHg Mean ± SD | 134.8±29.0 | 135.2±28.8 | -0.02 |  |
| HR at admission, bpm Mean ± SD | 81.2±20.6 | 81.2±19.8 | <0.001 |  |
| **Therapies on Admission** | | | | |
| GP IIb/IIIa inhibitors, % | 6.3 | 6.0 | 0.01 |  |
| Aspirin/P2Y_12_ inhibitors, % | 93.2 | 93.6 | -0.02 |  |
| **Medications during** **hospitalization** | | | | |
| ACE inhibitors/ARBs, % | 57.7 | 57.2 | 0.01 |  |
| Statins, % | 72.8 | 72.6 | 0.003 |  |
| β-blockers, % | 58.4 | 58.1 | 0.01 |  |
| **Outcomes** | | | ***P value*** |  |
| 30-day mortality, % | 16.4 | 12.9 |  |  |
| Risk Ratio (95% CI) | 1.32 (1.13 – 1.54) | | 0.0004 |  |
| Data are presented as percentages (%) ore mean ± SD, unless otherwise specified.  Abbreviations: ACE=angiotensin converting enzyme, angiotensin renin blocker, BMI=body mass index; CAD=coronary artery disease, CHD=coronary heart disease, CVD=cardiovascular disorders, ECG=electrocardiogram, GP=glycoprotein, HR=heart rate; PAD=peripheral artery disease, SBP=systolic blood pressure SMuRF: standard modifiable cardiovascular risk factor; | | | | |

| **Table S15.** Inverse probability weighting: Outcomes sorted by SMURFS status. Comparison between patients with diabetes as a solitary risk factor and those without any SMuRFs in the subgroup undergoing a**spirin and/or P2Y_12_ inhibitors use** | | | | |
| --- | --- | --- | --- | --- |
| **Characteristics** | **Diabetes (N=1496)** | **SMuRF-less**  **(N=8499)** | **Standardized difference** | |
| age, years Mean ± SD | 65.3±11.2 | 64.8±12.5 | 0.05 |  |
| **Cardiovascular risk factors** | | | | |
| Family history of CAD, % | 15.8 | 15.8 | 0.001 |  |
| Former smokers, % | 2.0 | 2.0 | -0.0002 |  |
| BMI ≥30 kg/m2, % | 13.2 | 13.4 | -0.01 |  |
| **Clinical history of CVD** | | | | |
| PAD, % | 1.6 | 1.6 | 0.003 |  |
| Prior stroke, % | 3.3 | 3.4 | -0.001 |  |
| **Clinical presentation on admission** | | | | |
| ST-segment shifts in anterior leads (at ECG), % | 20.7 | 20.1 | 0.02 |  |
| SBP at admission, mmHg Mean ± SD | 133.9±28.7 | 134.4±28.7 | -0.02 |  |
| HR at admission, bpm Mean ± SD | 81.2±19.9 | 80.9±19.7 | 0.01 |  |
| **Therapies on Admission** | | | | |
| GP IIb/IIIa inhibitors, % | 7.1 | 6.8 | 0.01 |  |
| Heparins (all), % | 87.4 | 87.4 | 0.0001 |  |
| **Medications during** **hospitalization** | | | | |
| ACE inhibitors/ARBs, % | 57.7 | 57.1 | 0.01 |  |
| Statins, % | 74.0 | 73.9 | 0.002 |  |
| β-blockers, % | 58.6 | 58.0 | 0.01 |  |
| **Outcomes** | | | ***P* value** |  |
| 30-day mortality, % | 15.4 | 12.0 | 0.0007 |  |
| Risk Ratio (95% CI) | 1.33 (1.14 – 1.56) | | 0.0003 |  |
| Data are presented as percentages (%) ore mean ± SD, unless otherwise specified.  Abbreviations: ACE=angiotensin converting enzyme, angiotensin renin blocker, BMI=body mass index; CAD=coronary artery disease, CHD=coronary heart disease, CVD=cardiovascular disorders, ECG=electrocardiogram, GP=glycoprotein, HR=heart rate; PAD=peripheral artery disease, SBP=systolic blood pressure; SMuRF: standard modifiable cardiovascular risk factor; | | | | |

| **Table S16 .** Inverse probability weighting: Outcomes stratified by SMURFS status. Comparison between patients with diabetes as a solitary risk factor and those without any SMuRFs in the subgroup administered Glycoprotein IIb/IIIa inhibitors. | | | | | |
| --- | --- | --- | --- | --- | --- |
| **Characteristics** | | **Diabetes (N=119)** | **SMuRF-less (N=754)** | **Standardized difference** | |
| Age, years Mean ± SD | | 64.8±11.5 | 64.6±12.6 | 0.02 |  |
| **Cardiovascular risk factors** | | | | | |
| Family history of CAD, % | | 12.2 | 14.7 | -0.07 |  |
| Former smokers, % | | 4.3 | 3.9 | 0.02 |  |
| BMI ≥30 kg/m2, % | | 13.9 | 15.1 | -0.04 |  |
| **Clinical history of CVD** | | | | | |
| PAD, % | | 1.0 | 0.9 | 0.01 |  |
| Prior stroke, % | | 2.5 | 2.4 | 0.004 |  |
| **Clinical presentation on admission** | | | | | |
| ST-segment shifts in anterior leads (at ECG), % | | 34.6 | 34.9 | -0.01 |  |
| SBP at admission, mmHg Mean ± SD | | 125.9±31.4 | 125.8±29.9 | 0.001 |  |
| HR at admission, bpm Mean ± SD | | 81.6±21.8 | 80.9±20.9 | 0.03 |  |
| **Therapies on Admission** | | | | | |
| Heparins, % | | 68.5 | 64.4 | 0.09 |  |
| Aspirin/ P2Y12 inhibitors, % | | 82.5 | 78.0 | 0.08 |  |
| **Medications during** **hospitalization** | | | | | |
| ACE inhibitors/ARBs, % | | 49.8 | 46.7 | 0.06 |  |
| Statins, % | | 60.8 | 58.0 | 0.06 |  |
| β-blockers, % | | 48.5 | 46.0 | 0.05 |  |
| **Outcomes** |  | | | ***P value*** |  |
| 30-day mortality, % | | 20.0 | 15.2 |  |  |
| Risk Ratio (95% CI) | | 1.39 (0.85 – 2.27) | | 0.19 |  |
| Data are presented as percentages (%) ore mean ± SD, unless otherwise specified.  Abbreviations: ACE=angiotensin converting enzyme, ARB=angiotensin renin blocker, BMI=body mass index; CAD=coronary artery disease, CHD=coronary heart disease, CVD=cardiovascular disorders, ECG=electrocardiogram, GP=glycoprotein, HR=heart rate; PAD=peripheral artery disease, SBP=systolic blood pressure. SMuRF: standard modifiable cardiovascular risk factor | | | | | |

| **Table S17.**Inverse probability weighting: outcomes stratified by sex and SMURFS status. Comparison between patients with current smoking as a solitary risk factor and those without any SMuRFs. | | | | | | | | |
| --- | --- | --- | --- | --- | --- | --- | --- | --- |
|  | **Women** | | | | **Men** | | | |
| **Characteristics** | **Current smoking (N=1088)** | **SMuRF-less (N=4039)** | **Standardized difference** | | **Current smoking (N=5137)** | **SMuRF-less**  **(N=7021)** | **Standardized difference** | |
| Age, years Mean ± SD | 64.2±12.7 | 64.5±13.5 | -0.02 |  | 59.4±11.9 | 59.3±13.2 | 0.009 |  |
| **Cardiovascular risk factors** | | | | |  |  |  |  |
| Family history of CAD, % | 19.4 | 18.7 | 0.02 |  | 19.8 | 19.7 | 0.001 |  |
| Former smokers, % | 0.0 | 0.5 | -0.09 |  | 0.0 | 0.6 | -0.06 |  |
| BMI ≥30 kg/m^2^, % | 11.9 | 11.8 | 0.001 |  | 14.1 | 14.2 | -0.0004 |  |
| **Clinical history of CVD** | | | | |  |  |  |  |
| PAD, % | 1.2 | 1.1 | 0.01 |  | 1.3 | 1.3 | 0.0001 |  |
| Prior stroke, % | 2.0 | 2.6 | -0.04 |  | 2.7 | 2.5 | 0.01 |  |
| **Clinical presentation on admission** | | | | |  |  |  |  |
| ST-segment shifts in anterior leads (at ECG), % | 17.7 | 18.5 | -0.02 |  | 21.1 | 21.2 | -0.0003 |  |
| SBP at admission, mmHg Mean ± SD | 130.4±26.7 | 130.4±30.1 | -0.001 |  | 132.9±27.9 | 132.9±28.9 | -0.002 |  |
| HR at admission, bpm Mean ± SD | 81.1±20.5 | 81.4±20.1 | -0.01 |  | 79.8±18.9 | 79.8±18.9 | -0.001 |  |
| **Outcomes** |  |  | ***P* value** |  |  |  | ***P* value** |  |
| 30-day mortality, % | 8.4 | 14.8 |  |  | 7.1 | 10.2 |  |  |
| Risk Ratio (95% CI) | 0.53 (0.42 – 0.67) | | <0.0001 |  | 0.68 (0.59 – 0.77) | | <0.0001 |  |
| Data are presented as percentages (%) or mean ±standard deviation, unless otherwise specified.  Abbreviations: BMI=body mass index; CAD=coronary artery disease, CHD=coronary heart disease, CVD=cardiovascular disorders, ECG=electrocardiogram, HR=heart rate; PAD=peripheral artery disease, SBP=systolic blood pressure; SMuRF: standard modifiable cardiovascular risk factor | | | | | | | | |

| **Table S18.** Inverse probability weighting: outcomes stratified by sex and SMURFS status. Comparison between patients with hypertension as a solitary risk factor and those without any SMuRFs. | | | | | | | | |
| --- | --- | --- | --- | --- | --- | --- | --- | --- |
|  | **Women** | | | | **Men** | | | |
| **Characteristics** | **Hypertension (N=4628)** | **SmuRF-less (N=4039)** | **Standardized difference** | | **Hypertension (N=5904)** | **SMuRF-less (N=7021)** | **Standardized difference** | |
| Mean ± SD age, years | 68.7±10.9 | 68.7±11.6 | 0.002 |  | 64.5±11.5 | 64.5±12.1 | 0.0003 |  |
| **Cardiovascular risk factors** | | | | |  |  |  |  |
| Family history of CAD, % | 18.2 | 18.0 | 0.004 |  | 17.3 | 17.3 | -0.002 |  |
| Former smokers, % | 0.8 | 0.7 | 0.01 |  | 3.2 | 3.3 | -0.001 |  |
| BMI ≥30 kg/m^2^, % | 13.8 | 14.0 | -0.01 |  | 15.9 | 16.1 | -0.01 |  |
| **Clinical history of CVD** | | | | |  |  |  |  |
| PAD, % | 1.2 | 1.1 | 0.003 |  | 1.6 | 1.5 | 0.004 |  |
| Prior stroke, % | 4.1 | 4.0 | 0.003 |  | 3.9 | 4.0 | -0.005 |  |
| **Clinical presentation on admission** | | | | |  |  |  |  |
| ST-segment shifts in anterior leads (at ECG), % | 18.6 | 18.7 | -0.003 |  | 19.6 | 20.0 | -0.01 |  |
| Mean ± SD SBP at admission, mmHg | 136.1±29.7 | 136.5±31.5 | -0.01 |  | 137.6±28.6 | 137.9±30.7 | -0.01 |  |
| Mean ± SD HR at admission, bpm | 82.3±19.8 | 82.4±21.1 | -0.004 |  | 80.9±19.5 | 80.9±19.9 | -0.004 |  |
| **Outcomes** |  |  | ***P* value** |  |  |  | ***P* value** |  |
| 30-day mortality, % | 13.6 | 15.5 |  |  | 8.6 | 11.0 |  |  |
| Risk Ratio (95% CI) | 0.86 (0.76 – 0.97) | | 0.01 |  | 0.77 (0.68 – 0.86) | | <0.0001 |  |
| Data are presented as percentages (%) or mean ±standard deviation, unless otherwise specified.  Abbreviations: BMI=body mass index; CAD=coronary artery disease, CHD=coronary heart disease; CVD=cardiovascular disorders, ECG=electrocardiogram, HR=heart rate; PAD=peripheral artery disease, SBP=systolic blood pressure; SMuRF: standard modifiable cardiovascular risk factor; | | | | | | | | |

| **Table S19.** Inverse probability weighting: Outcomes stratified by sex and SMURFS status. Comparison between patients with hypercholesterolemia as a solitary risk factor and those without any SMuRFs. | | | | | | | | |
| --- | --- | --- | --- | --- | --- | --- | --- | --- |
|  | **Women** | | | | **Men** | | | |
| **Characteristics** | **Hypercholesterolemia (N=755)** | **SMuRF-less (N=4039)** | **Standardized difference** | | **Hypercholesterolemia (N=1439)** | **SMuRF-less (N=7021)** | **Standardized difference** | |
| Age, years Mean ± SD | 66.7±11.7 | 66.7±12.2 | -0.003 |  | 62.2±12.2 | 62.3±12.3 | -0.01 |  |
| **Cardiovascular risk factors** | | | | |  |  |  |  |
| Family history of CAD, % | 17.9 | 18.0 | -0.002 |  | 17.5 | 17.1 | 0.01 |  |
| Former smokers, % | 0.6 | 0.7 | -0.01 |  | 2.6 | 2.5 | 0.003 |  |
| BMI ≥30 kg/m^2^, % | 13.0 | 12.6 | 0.01 |  | 15.3 | 14.8 | 0.01 |  |
| **Clinical history of CVD** | | | | |  |  |  |  |
| PAD, % | 1.2 | 1.2 | -0.01 |  | 1.4 | 1.4 | -0.0004 |  |
| Prior stroke, % | 3.0 | 2.9 | 0.01 |  | 3.2 | 3.0 | 0.01 |  |
| **Clinical presentation on admission** | | | | |  |  |  |  |
| ST-segment shifts in anterior leads (at ECG), % | 17.0 | 17.9 | -0.02 |  | 20.2 | 20.1 | 0.004 |  |
| SBP at admission Mean ± SD Mean ± SD | 129.9±27.8 | 129.9±30.1 | 0.001 |  | 131.9±26.9 | 131.9±29.2 | -0.0001 |  |
| HR at admission, bpm Mean ± SD | 81.4±20.3 | 81.2±20.1 | 0.01 |  | 79.2±18.3 | 79.6±18.9 | -0.02 |  |
| **Outcomes** |  |  | ***P* value** |  |  |  | ***P* value** |  |
| 30-day mortality, % | 10.5 | 16.0 |  |  | 5.6 | 11.4 |  |  |
| Risk Ratio (95% CI) | 0.62 (0.48 – 0.79) | | <0.0001 |  | 0.46 (0.37 – 0.59) | | <0.0001 |  |
| Data are presented as percentages (%) or mean ±standard deviation, unless otherwise specified.  Abbreviations: BMI=body mass index; CAD=coronary artery disease, CHD=coronary heart disease, CVD=cardiovascular disorders, ECG=electrocardiogram, HR=heart rate; PAD=peripheral artery disease, SBP=systolic blood pressure; SMuRF: standard modifiable cardiovascular risk factor; | | | | | | | | |

| **Table S20**. Inverse probability weighting: outcomes stratified by sex and SMURFS status. Comparison between patients with both diabetes and current smoking as concurrent risk factors and those without any SMuRFs | | | | | | | | | |
| --- | --- | --- | --- | --- | --- | --- | --- | --- | --- |
|  | ***Women*** | | | | | ***Men*** | | | |
| **Characteristics** | **Diabetes and current smoking (N=143)** | | **SMuRF-less**  **(N=4039)** | **Standardized difference** | | **Diabetes and current smoking (N=595)** | **SMuRF-less (N=7021)** | **Standardized difference** | |
| Age, years Mean ± SD | 66.8±11.7 | | 66.9±12.1 | -0.01 |  | 61.4±10.1 | 62.5±12.3 | -0.09 |  |
| **Cardiovascular risk factors** | | | | |  |  |  |  |  |
| Family history of CAD, % | 16.2 | | 15.4 | 0.02 |  | 15.1 | 15.0 | 0.01 |  |
| Former smokers, % | 0.0 | | 0.6 | -0.06 |  | 0.0 | 1.1 | -0.09 |  |
| BMI ≥30 kg/m^2^, % | 11.1 | | 11.7 | -0.02 |  | 14.0 | 14.3 | -0.01 |  |
| **Clinical history of CVD** |  | |  |  |  |  |  |  |  |
| PAD, % | 0.8 | | 1.1 | -0.03 |  | 1.4 | 1.4 | -0.004 |  |
| Prior stroke, % | 2.5 | | 3.0 | -0.03 |  | 2.4 | 2.9 | -0.03 |  |
| **Clinical presentation on admission** | | | | |  |  |  |  |  |
| ST-segment shifts in anterior leads (at ECG), % | 19.4 | | 18.1 | 0.03 |  | 19.3 | 19.6 | -0.008 |  |
| SBP at admission, mmHg Mean ± SD | 130.7±32.0 | | 129.5±30.1 | 0.04 |  | 132.3±26.9 | 132.1±29.2 | 0.01 |  |
| HR at admission, bpm Mean ± SD | 83.8±18.6 | | 82.5±20.3 | 0.08 |  | 79.8±19.8 | 80.0±19.3 | -0.01 |  |
| **Outcomes** | |  | | ***P* value** |  |  |  | ***P* value** |  |
| 30-day mortality, % | 21.2 | | 16.2 |  |  | 10.4 | 11.5 |  |  |
| Risk Ratio (95% CI) | 1.39 (0.92 – 2.09) | | | 0.12 |  | 0.89 (0.68 – 1.17) | | 0.40 |  |
| Data are presented as percentages (%) or mean ±standard deviation, unless otherwise specified.  Abbreviations: BMI=body mass index; CAD=coronary artery disease, CHD=coronary heart disease, CVD=cardiovascular disorders, ECG=electrocardiogram, HR=heart rate; PAD=peripheral artery disease, SBP=systolic blood pressure; SMuRF: standard modifiable cardiovascular risk factor; | | | | | | | | | |

| **Table S21.** Inverse probability weighting: outcomes stratified by sex and SMURFS status. Comparison between patients with both diabetes and hypercholesterolemia as concurrent risk factors and those without any SMuRFs | | | | | | | | | |
| --- | --- | --- | --- | --- | --- | --- | --- | --- | --- |
|  | | ***Women*** | | | | ***Men*** | | | |
| **Characteristics** | | **Diabetes and hypercholesterolemia** **(N=310)** | **SMuRF-less**  **(N=4039)** | **Standardized difference** | | **Diabetes and hypercholesterolemia** **(N=396)** | **SMuRF-less**  **(N=7021)** | **Standardized difference** | |
| Age, years Mean ± SD | | 66.9±9.7 | 67.1±12.0 | -0.01 |  | 62.7±10.9 | 62.8±12.2 | -0.01 |  |
| **Cardiovascular risk factors** | | | | | |  |  |  |  |
| Family history of CAD, % | | 15.8 | 15.8 | <0.0001 |  | 14.7 | 15.0 | -0.01 |  |
| Former smokers, % | | 0.7 | 0.6 | 0.02 |  | 2.6 | 2.4 | 0.01 |  |
| BMI ≥30 kg/m^2^, % | | 12.1 | 12.0 | 0.003 |  | 14.8 | 14.3 | 0.01 |  |
| **Clinical history of CVD** | | | | | |  |  |  |  |
| PAD, % | | 1.1 | 1.2 | -0.01 |  | 1.2 | 1.3 | -0.01 |  |
| Prior stroke, % | | 2.8 | 3.0 | -0.01 |  | 3.0 | 2.9 | 0.01 |  |
| **Clinical presentation on admission** | | | | | |  |  |  |  |
| ST-segment shifts in anterior leads (at ECG), % | | 18.0 | 18.0 | 0.001 |  | 19.6 | 19.6 | 0.0003 |  |
| SBP at admission, mmHg Mean ± SD | | 129.1±26.8 | 129.4±30.1 | -0.01 |  | 132.3±27.8 | 131.8±29.2 | 0.02 |  |
| HR at admission, bpm Mean ± SD | | 81.7±20.7 | 81.6±20.4 | 0.01 | 0 | 79.9±19.6 | 79.9±19.2 | 0.0001 |  |
| **Outcomes** |  | | | ***P* value** | |  |  | ***P* value** |  |
| 30-day mortality, % | | 15.1 | 16.4 |  |  | 9.0 | 11.6 |  |  |
| Risk Ratio (95% CI) | | 0.91 (0.66 – 1.25) | | 0.56 |  | 0.75 (0.53 – 1.06) | | 0.11 |  |
| Data are presented as percentages (%) or mean ±standard deviation, unless otherwise specified.  Abbreviations: BMI=body mass index; CAD=coronary artery disease, CHD=coronary heart disease, CVD=cardiovascular disorders, ECG=electrocardiogram, HR=heart rate; PAD=peripheral artery disease, SBP=systolic blood pressure. SMuRF: standard modifiable cardiovascular risk factor; | | | | | | | | | |

| **Table S22.** Inverse probability weighting: Comparison between patients with both diabetes and hypertension as concurrent risk factors and those without any SMuRFs | | | | | | | | |
| --- | --- | --- | --- | --- | --- | --- | --- | --- |
|  | ***Women*** | | | | ***Men*** | | | |
| **Characteristics** | **Diabetes and hypertension (N=1915)** | **SMuRF-less**  **(N=4039)** | **Standardized difference** | | **Diabetes and hypertension (N=1825)** | **SMuRF-less**  **(N=7021)** | **Standardized difference** | |
| Age, years Mean ± SD | 69.0±9.5 | 68.5±11.7 | 0.05 |  | 64.5±10.4 | 63.8±12.2 | 0.06 |  |
| **Cardiovascular risk factors** | | | | |  |  |  |  |
| Family history of CAD, % | 17.5 | 17.4 | 0.004 |  | 15.0 | 15.5 | -0.01 |  |
| Former smokers, % | 0.7 | 0.7 | 0.001 |  | 2.8 | 3.0 | -0.01 |  |
| BMI ≥30 kg/m^2^, % | 14.7 | 14.9 | -0.01 |  | 15.5 | 16.1 | -0.02 |  |
| **Clinical history of CVD** | | | | |  |  |  |  |
| PAD, % | 1.8 | 1.7 | 0.01 |  | 1.9 | 1.8 | 0.004 |  |
| Prior stroke, % | 3.8 | 3.8 | -0.003 |  | 3.7 | 3.5 | 0.01 |  |
| **Clinical presentation on admission** | | | | |  |  |  |  |
| ST-segment shifts in anterior leads (at ECG), % | 19.3 | 19.3 | -0.0001 |  | 19.8 | 19.8 | 0.0002 |  |
| SBP at admission, mmHg Mean ± SD | 132.5±30.9 | 133.3±31.3 | -0.03 |  | 134.1±28.6 | 134.2±29.9 | -0.01 |  |
| HR at admission, bpm Mean ± SD | 82.7±21.3 | 82.9±21.8 | -0.01 |  | 80.9±20.3 | 81.1±20.4 | -0.01 |  |
| **Outcomes** | | | ***P* value** |  |  |  | ***P* value** |  |
| 30-day mortality, % | 18.5 | 16.6 |  |  | 13.0 | 11.7 |  |  |
| Risk Ratio (95% CI) | 1.14 (0.99 – 1.32) | | 0.07 |  | 1.12 (0.96 – 1.31) | | 0.14 |  |
| Data are presented as percentages (%) or mean ±standard deviation, unless otherwise specified.  Abbreviations: BMI=body mass index; CAD=coronary artery disease, CHD=coronary heart disease, CVD=cardiovascular disorders, ECG=electrocardiogram, HR=heart rate; PAD=peripheral artery disease, SBP=systolic blood pressure SMuRF: standard modifiable cardiovascular risk factor; | | | | | | | | |

| **Table S23. Interaction Test of diabetes-related mortality across additional SMuRF combinations** | | | | |
| --- | --- | --- | --- | --- |
| **Sex** | **Comparison** | **Ratio of RRs** | **95 % CI** | **P value** |
| Men | Diabetes + Hypertension vs Diabetes only | 1.25 | 0.99–1.60 | 0.036 |
| Men | Diabetes + Smoking vs Diabetes only | 1.58 | 1.13–2.19 | 0.004 |
| Men | Diabetes + Hypercholesterolemia. vs Diabetes only | 1.87 | 1.26 – 2.77 | <0.001 |
| Women | Diabetes + Hypertension vs Diabetes only | 1.13 | 0.83–1.44 | 0.13 |
| Women | Diabetes + Smoking vs Diabetes only | 0.93 | 0.59–1.46 | 0.64 |
| Women | Diabetes + Hypercholesterolemia. vs Diabetes only | 1.42 | 0.97-2.06 | 0.04 |
| Ratios of relative risks (RRs) for 30-day mortality comparing each “Diabetes + SMuRF” subgroup versus patients with Diabetes Only, derived using the Altman test of interaction (BMJ 2003; 326:219). Ratios > 1 indicate attenuation of diabetes-related risk when additional SMuRFs are present | | | | |

**REFERENCES**

1. Bugiardini R, Badimon L, Investigators I-T, Coordinators. The International Survey of Acute Coronary Syndromes in Transitional Countries (ISACS-TC): 2010-2015. Int J Cardiol 2016;217 Suppl:S1-6.
2. Vasic A, Vasiljevic Z, Mickovski-Katalina N, Mandic-Rajcevic S, Soldatovic I. Temporal Trends in Acute Coronary Syndrome Mortality in Serbia in 2005-2019: An Age-Period-Cohort Analysis Using Data from the Serbian Acute Coronary Syndrome Registry (RAACS). Int J Environ Res Public Health 2022;19.
3. van Buuren, S, Groothuis-Oudshoorn K. "mice: Multivariate imputation by chained equations in R." Journal of Statistical Software. 2011;45(3). doi:10.18637/jss.v045.i03
4. Austin PC, Stuart EA. Moving towards best practice when using inverse probability of treatment weighting (IPTW) using the propensity score to estimate causal treatment effects in observational studies. *Stat Med* 2015; 34(28): 3661-79.
5. Katz D, Baptista J, Azen SP, et al. Obtaining Confidence Intervals for the Risk Ratio in Cohort Studies. *Biometrics* 1978;34(3):469-74. doi: 10.2307/2530610
6. Dongsheng Y, Dalton JE. A unified approach to measuring the effect size between two groups using SAS®. SAS Global Forum. Vol. 335. 2012.
7. Altman DG, Bland JM. Interaction revisited: the difference between two estimates. BMJ. Jan 25 2003;326(7382):219. doi:10.1136/bmj.326.7382.219
